# Notch signalling regulates epibranchial placode patterning and segregation

**DOI:** 10.1242/dev.183665

**Published:** 2020-02-17

**Authors:** Li Wang, Junjie Xie, Haoran Zhang, Long Hin Tsang, Sze Lan Tsang, Eike-Benjamin Braune, Urban Lendahl, Mai Har Sham

**Affiliations:** 1 School of Biomedical Sciences, LKS Faculty of Medicine, The University of Hong Kong, Pokfulam, Hong Kong SAR, China; 2 Department of Cell and Molecular Biology, Karolinska Institutet, Stockholm, SE-171 77, Sweden

**Keywords:** Epibranchial placode, Pharyngeal ectoderm, Notch signalling, N1ICD, Rbpj, Mouse

## Abstract

Epibranchial placodes are the geniculate, petrosal and nodose placodes that generate parts of cranial nerves VII, IX and X, respectively. How the three spatially separated placodes are derived from the common posterior placodal area is poorly understood. Here, we reveal that the broad posterior placode area is first patterned into a *Vgll2^+^/Irx5^+^* rostral domain and a *Sox2^+^/Fgf3^+^/Etv5^+^* caudal domain relative to the first pharyngeal cleft. This initial rostral and caudal patterning is then sequentially repeated along each pharyngeal cleft for each epibranchial placode. The caudal domains give rise to the neuronal and non-neuronal cells in the placode, whereas the rostral domains are previously unrecognized structures, serving as spacers between the final placodes. Notch signalling regulates the balance between the rostral and caudal domains: high levels of Notch signalling expand the caudal domain at the expense of the rostral domain, whereas loss of Notch signalling produces the converse phenotype. Collectively, these data unravel a new patterning principle for the early phases of epibranchial placode development and a role for Notch signalling in orchestrating epibranchial placode segregation and differentiation.

## Abstract (precis)

**Summary:** Notch signalling regulates the rostral-caudal patterning and commitment of the epibranchial placodal region into repeated *Vgll2^+^/Irx5^+^* and *Sox2^+^/Fgf3^+^/Etv5^+^* domains along the pharyngeal clefts, leading to the formation of individual placodes.

## INTRODUCTION

Cranial placodes are transient ectodermal thickenings that give rise to specialized sensory organs and ganglia of the cephalic peripheral nervous system. Cranial placodes arise from a common pre-placodal region, which separates into anterior, intermediate and posterior placodal areas (PPA), before segregating into discrete individual placodes (Saint-Jeannet and Moody, 2014; Schlosser, 2010; Schlosser and Ahrens, 2004; Streit, 2004). The epibranchial placodes are named based on their locations dorsal-caudal to the pharyngeal (branchial) clefts and stem from the PPA. They give rise to the viscerosensory neurons of the distal ganglia of the facial (VIII), glossopharyngeal (IX) and vagal (X) cranial nerves to innervate various visceral organs to enable them to collect sensory information (Baker and Bronner-Fraser, 2001).

**Fig. 1.**
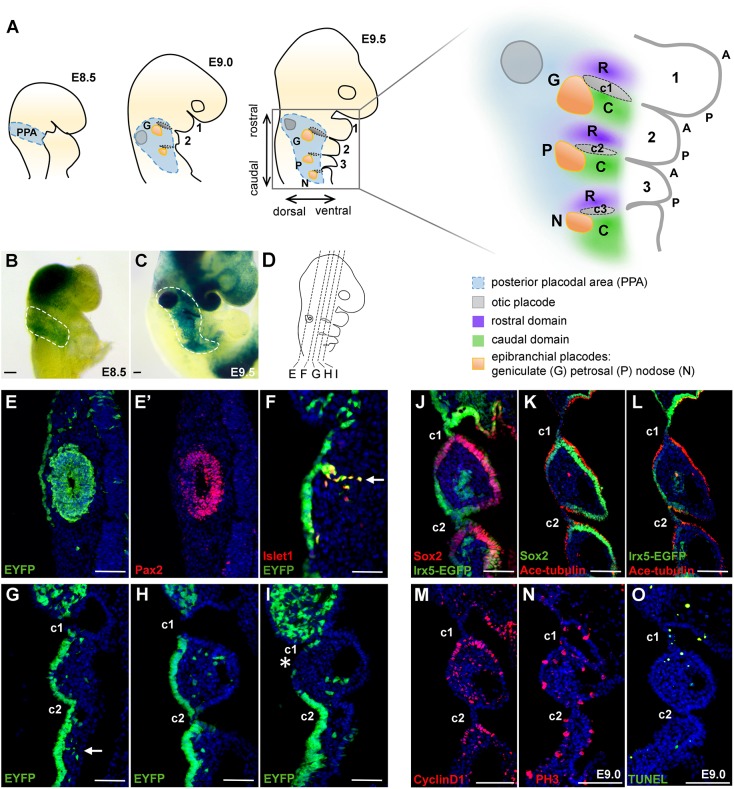
**Analysis of epibranchial placodal cells derived from the posterior placodal area.** (A) Schematic illustrating the locations of the posterior placodal area, the otic and epibranchial placode territories at E8.5, E9.0 and E9.5. The alignment of epibranchial placodal domains (rostral and caudal to each cleft) with the pharyngeal arches at E9.5 is illustrated. (B,C) Whole-mount *lacZ* reporter-stained *Pax2Cre; R26R^lacZ^* embryos at (B) E8.5 (*n*=3) and (C) E9.5 (*n*=3). The dashed lines illustrate the PPA domain at E8.5 and otic/epibranchial domains at E9.5. (D) Schematic illustrating the plane of sections for panels E-I (dashed lines). (E-I) Immunostaining for EYFP, Pax2 and Islet1 in serial coronal sections of an E9.5 *Pax2Cre; R26R^EYFP^* embryo from dorsal to ventral (*n*=3). Arrows indicate positions of the delaminating neurons from geniculate (F) and petrosal (G) placodes. Asterisk (I) indicates the distal pharyngeal ectodermal cells. (J) Co-immunostaining of EGFP and Sox2 on E9.5 *Irx5^EGFP/+^* coronal sections (*n*=3). (K) Co-immunostaining of Sox2 and acetylated tubulin on coronal section of E9.5 WT embryos (*n*=3). (L) Co-immunostaining of EGFP and acetylated tubulin on a coronal section of E9.5 *Irx5^EGFP/+^* embryos (*n*=3). (M) Immunostaining of cyclin D1 on coronal sections of E9.5 WT embryos (*n*=3). (N,O) Immunostaining of phospho-histone H3 (PH3) (N) and TUNEL (O) on coronal sections of E9.0 WT embryos (*n*=3). Scale bars: 100 µm. 1, 2, 3 indicate the first, second and third pharyngeal arches; c1, c2, c3 indicate the first, second and third pharyngeal clefts; A, anterior; C, caudal; P, posterior; R, rostral.

The PPA emerges on the ectodermal surface adjacent to the neural plate, at the level of the caudal hindbrain and rostral to the first somite. The PPA contains a common otic-epibranchial precursor domain that expresses *Pax2*, *Sox2* and *Sox3* and is recognizable at around embryonic day (E) 8.5 (4-6 somite stage, ss) in mouse and chick embryos (Fig. 1A) (Chen and Streit, 2013). The otic territory and the lateral epibranchial territory, a contiguous region of thickened epithelium on the proximal pharyngeal ectoderm, become molecularly distinct and gradually separated by E9.0 (14-16 ss). At around E9.25 (20 ss), the otic placode invaginates to form the otic cup, and the epibranchial territory is split into a rostral geniculate domain, and a caudal domain, which will become the future petrosal and nodose placodes (Ishii et al., 2001; Ladher et al., 2010; Washausen and Knabe, 2017). By E9.5 (24-27 ss), the otic vesicle is formed, and the three pairs of epibranchial placodes are confined to the dorsal-caudal position of their respective pharyngeal clefts (Fig. 1A). Although it has long been established that the *Neurog2*-expressing domain of the epibranchial placodes generates neurons of the cranial nerves, it was recently observed that each epibranchial placode also harbours a non-neuronal cell population, characterized by combined *Sox2* and *Fgf3* expression (Zhang et al., 2017).

During the early stages of placode development, the PPA contains multipotent precursors that acquire different placodal fates. For the epibranchial placodes, it remains unclear whether the PPA contains predetermined precursors that are intermingled and subsequently segregate into distinct placodes, or whether specification of regional placodal fates occurs later when uncommitted cells respond to local signalling factors. A key feature of epibranchial placodes is their coordinated development with the pharyngeal arches (PAs). The three pairs of epibranchial placodes are intimately associated with the pharyngeal arch segmentation process, during which the pharyngeal ectoderm fuses with the endoderm forming clefts on the surface and the pocket-like pouches in the endoderm (Graham, 2003). The morphological changes and cell movements during the formation of PAs may separate the pool of epibranchial placodal precursors into their final locations. *Pax2*, *Sox2* and *Sox3* are initially broadly expressed in the PPA and retained in the placodal cells, but downregulated in the ‘interplacodal’ regions (Ishii et al., 2001; Tripathi et al., 2009; Washausen and Knabe, 2017). It has been suggested that apoptosis in the ‘interplacodal’ regions and proliferation of placodal cells could promote the physical segregation of the three discrete epibranchial placodes (Washausen and Knabe, 2013, 2017, 2018; Washausen et al., 2005). The mechanisms underlying the segregation of the placodal precursors into specific epibranchial placodes, however, remain unclear.

A number of signalling mechanisms are implicated in epibranchial placode differentiation, including BMP, Wnt, FGF and Notch signalling (Begbie et al., 2002, 1999; Freter et al., 2008; Koo et al., 2005; Kriebitz et al., 2009; Litsiou et al., 2005; McCarroll and Nechiporuk, 2013; Urness et al., 2010). The Notch signalling pathway is an evolutionarily conserved cell-cell communication system that regulates cell differentiation and homeostasis in most organs (Siebel and Lendahl, 2017). In mammals, there are four receptors (Notch1-4) and five ligands (Jag1-2 and Dll1,3,4). Interaction between transmembrane Notch ligands and receptors on juxtaposed cells initiates signalling in the receptor-expressing cell. Ligand interaction leads to sequential proteolytic cleavage of the Notch receptor, which liberates its C-terminal domain (referred to as the Notch intracellular domain, NICD). NICD translocates to the nucleus to form a ternary transcriptional complex with the DNA-binding protein CSL (RBP-j κ) and MAML. When Notch signalling is not activated, in the absence of NICD, CSL acts as a repressor by interacting with co-repressors, but switches to an activator when NICD contacts CSL to displace the co-repressors with co-activators (Bray, 2016; Siebel and Lendahl, 2017). Notch receptors are also modified by Fringe proteins, which are glycosyltransferases, and Fringe-mediated extensions of O-linked fucose-adducts on the Notch receptor extracellular domain alter the receptor's preference for signalling via Dll and Jagged types of ligand (for a review, see Harvey and Haltiwanger, 2018). Notch signalling in conjunction with Eya1 has recently been shown to be important for differentiation to the neuronal (*Neurog2^+^*) and non-neuronal (*Sox2^+^/Fgf3^+^*) fates in epibranchial placodes (Zhang et al., 2017).

In this article, we address the early steps of epibranchial placode development to elucidate how cell specification occurs from the PPA to the segregation of the spatially separated geniculate, petrosal and nodose epibranchial placodes. We identify an early patterning event, with the appearance of a rostral *Vgll2^+^/Irx5^+^* domain and a caudal *Sox2^+^/Fgf3^+^/Etv5^+^* domain located on opposite sides of the first pharyngeal cleft. This rostral-caudal patterning is then repeated along the second and third clefts, which precede the formation of the geniculate, petrosal and nodose epibranchial placodes. Notch signalling coordinates the balance between the rostral and caudal domains: high levels of Notch promote the caudal programme, whereas loss of Notch signalling activity conversely expands the rostral territory. In conclusion, these data provide novel insights into the genesis of epibranchial placodes and define a role for Notch signalling in epibranchial patterning and segregation.

## RESULTS

### A *Pax2^+^* posterior placodal area gives rise to multiple placodal and epithelial cell types

*Pax2* is one of the earliest specific makers for the PPA (Baker et al., 2008; McCarroll et al., 2012; Ohyama and Groves, 2004a; Streit, 2002). To follow the fate of the *Pax2^+^* PPA cells, we performed lineage-tracing experiments using *Pax2-Cre* (Ohyama and Groves, 2004a) and *Rosa26-EYFP* (Srinivas et al., 2001) or *Rosa26-lacZ* (Soriano, 1999) mice. At E8.5, *lacZ* reporter expression was observed at the PPA, covering a lateral surface caudal to the first pharyngeal arch (PA1) and rostral to the first somite (Fig. 1B), a distribution consistent with previous data from both mouse and chick embryos (Wright and Mansour, 2003). At this stage, other *Pax2^+^* cells can also be found in the brain and the migrating neural crest within the first pharyngeal arch. At E9.5, *lacZ* reporter expression in the pharyngeal region covered a broad domain encompassing the otic vesicle and proximal pharyngeal ectoderm (Fig. 1C). Analysis of serial coronal sections of E9.5 *Pax2-Cre;Rosa^EYFP^* embryos revealed that the otic vesicle, the geniculate and petrosal placodal cells as well as delaminated Islet1 (Isl1)^+^ neuroblasts were labelled by EYFP (Fig. 1E-I). Notably, at this stage *Pax2* expression was confined to the otic vesicle (Fig. 1E′) and *Pax2* was no longer expressed in the epibranchial epithelial cells. Serial section analysis showed that the proximal pharyngeal epithelial cells (Fig. 1E-H), but not the cells covering the distal pharyngeal arch, were EYFP^+^ (Fig. 1I), indicating that the whole proximal pharyngeal ectodermal area is derived from the *Pax2^+^* PPA. Similar results were also obtained using *Sox2CreERT2;Rosa^EYFP^* mice, showing that a broad range of pharyngeal epithelial cells were derived from the PPA (Fig. S1; Fig. 5A,C,E).

Within the *Pax2*-labelled proximal pharyngeal ectoderm, the epibranchial placodes could be identified as thickened epithelial cells, whereas the surrounding interplacodal pharyngeal ectoderm thinned out and adopted a surface epithelial morphology (Müller and O'Rahilly, 1988; Tripathi et al., 2009; Washausen et al., 2005). We further examined the epibranchial placodal area using *Sox2*, a placodal marker gene; *Irx5*, which is expressed in PPA (Feijoo et al., 2009; Glavic et al., 2004); and acetylated tubulin, which marks motile cilia on ciliated cells. We found that the epibranchial placodal cells expressing *Sox2* were marked with acetylated tubulin (Fig. 1J,K). Conversely, the epithelial cells marked by *Irx5* (indicated by EGFP in *Irx5^EGFP/+^* embryos) had a much lower density of acetylated tubulin (Fig. 1L). The Sox2^+^ epibranchial placodal cells also expressed cyclin D1 (Fig. 1M), suggesting that these cells were proliferative. We further examined cell proliferation and apoptosis by phospho-histone H3 (PH3) staining and terminal deoxynucleotidyl transferase dUTP nick end labelling (TUNEL) analysis, respectively (Fig. 1N,O; Fig. S3). Our data confirmed previous findings that apoptosis could be detected in the pharyngeal clefts, whereas epithelial regions were proliferative (Washausen et al., 2005). These results are consistent with the notion that *Pax2^+^* PPA progenitors gave rise to both otic and epibranchial placodes, as well as their surrounding non-neural cells (as indicated in Fig. 1A) (Ohyama and Groves, 2004b; Streit, 2002).

### Stepwise regionalization of epibranchial placode and proximal pharyngeal ectoderm

We next addressed the question of how the pool of *Pax2^+^* PPA progenitors may segregate into three discrete epibranchial placodes as well as interplacodal pharyngeal ectodermal cells. We first investigated the spatiotemporal patterning of the epibranchial placodes from E8.5 to E9.5 using a series of placodal and pharyngeal markers including *Eya1* and *Six1* (placodal progenitor markers); *Neurog2* (the earliest pre-neural marker in epibranchial placode; Fode et al., 1998); *Sox2* (essential for epibranchial neural competence; Gou et al., 2018; Tripathi et al., 2009); *Irx5* (expressed in PPA; Feijoo et al., 2009; Glavic et al., 2004); vestigial-like2 (*Vgll2*) (expressed in the pharyngeal region; Chen et al., 2017; Johnson et al., 2011); *Fgf3*; and the FGF downstream target *Etv5* (essential for pharyngeal morphogenesis) (Urness et al., 2011).

**Fig. 2.**
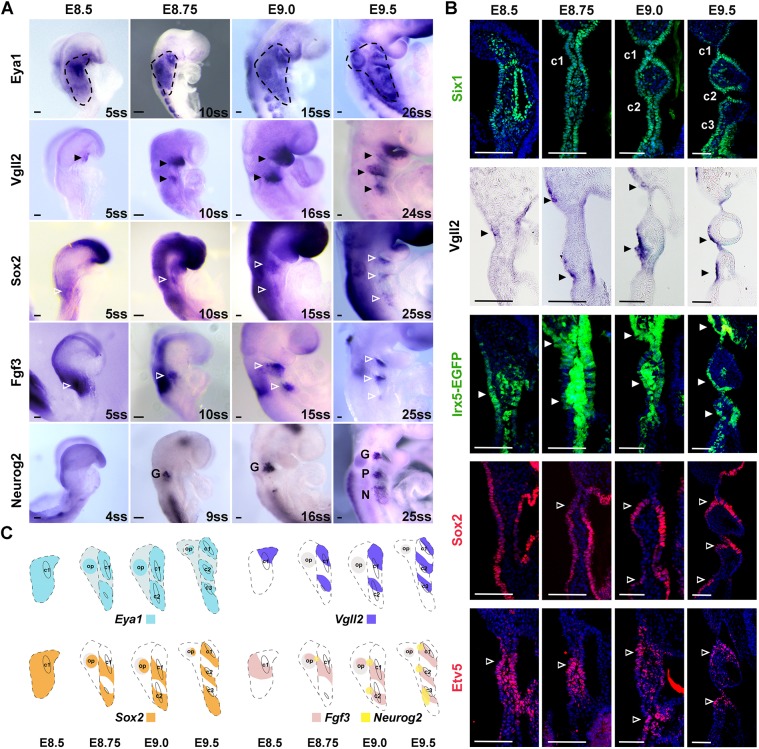
**Spatiotemporal gene expression patterns during rostral and caudal regionalization of proximal pharyngeal ectoderm.** (A) Whole-mount *in situ* hybridization of WT embryos from E8.5 to E9.5 with somite stages (ss) and genes as indicated (*n*≥3 for each stage and probe). The regions circled by black dashed lines indicate the *Eya1* expression domain. (B) Immunostaining for Six1, Etv5 and Sox2 on coronal sections of WT embryos, immunostaining for EGFP on coronal sections of *Irx5^EGFP/+^* embryos, and whole-mount *in situ* hybridization for *Vgll2* in coronal sections of embryos from E8.5 to E9.5. In A and B, filled arrowheads indicate expression of *Vgll2* (black) and Irx5 (white) in domains rostral to the clefts, and unfilled arrowheads indicate expression of Sox2, Fgf3 and Etv5 in domains caudal to the clefts. Scale bars: 100 µm. (C) Schematics illustrating the expression regions of *Eya1* (circled by dashed lines, blue), *Vgll2* (purple), *Sox2* (orange), *Fgf3* (pink) and *Neurog2* (yellow) during the specification of epibranchial placodes at the indicated stages. c1, c2, c3 indicate the first, second and third pharyngeal clefts; G, geniculate placode; N, nodose placode; op, otic placode; P, petrosal placode.

At around E8.5, before the appearance of the first pharyngeal cleft (c1), *Eya1* was detected specifically in the PPA (Fig. 2A). At this stage, *Vgll2* expression first appeared in the emerging PA1 and *Fgf3* was expressed in the hindbrain, but neither of them was detected in the epibranchial placodal region (Fig. 2A) (see also Zhang et al., 2017). At around E8.75, c1 became morphologically visible whereas c2 (second pharyngeal cleft) was not yet formed. *Eya1* was expressed in the expanded PPA, spanning from the rostral part of the first cleft, caudally extending to the first somite, dorsally to the otic placodal region and ventrally to the proximal PA (Fig. 2A). *Vgll2* was expressed rostrally of c1 and at the presumptive c2, and its expression in PA1 (beyond the epibranchial placodal region) was expanded, whereas *Fgf3* was first detected at a position caudal of c1 (Fig. 2A). At around E9.0, c2 could be morphologically identified ventral to the otic pit. *Eya1* continued to label the broad epibranchial placodal region, and a second *Fgf3^+^* domain appeared (Fig. 2A). The locations of the *Fgf3*^+^ domains were caudal and complementary to the *Vgll2*^+^ domains. At E9.5, all three clefts were formed. *Eya1* expression persisted in the whole otic-epibranchial placodal region, with distinctive expression in the otic vesicle and geniculate, petrosal and nodose placodes, and a lower level of expression at the interplacodal pharyngeal epithelium. Three *Vgll2^+^* and three *Fgf3^+^* domains were detected at the proximal pharyngeal arches in a complementary manner (Fig. 2A). *Sox2* expression appeared earlier than *Fgf3* in the PPA at E8.5 and was gradually confined to the domain caudal of each cleft until E9.5 (Fig. 2A,B). *Neurog2* expression was not detected until E8.75, when it was found specifically at the dorsal end of each *Fgf3^+^* domain (Fig. 2A) (see also Zhang et al., 2017). As illustrated in Fig. 1A, ‘rostral’ and ‘caudal’ domains are in relation to the pharyngeal clefts.

To examine gene expression patterns across the embryo, coronal sections of embryos from E8.5 to E9.5 were analysed with different markers. *Six1*, as one of the pan-placodal markers, was expressed early at the placodal ectoderm, and also in the pharyngeal endodermal, mesenchymal and mesodermal cells at E8.5 (Fig. 2B). Later, in line with the development of pharyngeal arches from E8.75 to E9.5, three clefts and pouches appeared sequentially. *Six1* expression was broadly detected at the epibranchial placodes and proximal PA ectodermal cells (Fig. 2B). Sox2 was initially expressed broadly along the PPA ectoderm at E8.5, but it was gradually restricted to domains caudal to each cleft from E9.0 (Fig. 2B). Etv5 (also called Erm), a downstream target of FGF signalling (Roehl and Nüsslein-Volhard, 2001), was expressed in the pharyngeal ectoderm, endoderm and mesenchymal cells. From E9.0, Etv5 was expressed at positions caudal to each of the developing pharyngeal clefts (Fig. 2B). In contrast, Irx5 was highly expressed in the pharyngeal mesoderm, but by E9.5 its expression was observed in the ectodermal region rostral to each cleft (Fig. 2B). Similarly, *Vgll2* was expressed only at rostral domain of each cleft as shown in coronal sections (Fig. 2B), complementary to the Sox2^+^/Etv5^+^ regions along the proximal ectoderm.

In summary, our results suggest that, after separation from the otic territory, the *Eya1^+^/Six1^+^* epibranchial placodal area expanded caudally as the pharyngeal arches were generated. From E8.5 onwards, accompanying the appearance of each pharyngeal cleft, a *Vgll2^+^/Irx5^+^* domain (rostral to each cleft) and a *Sox2^+^/Etv5^+^* domain (caudal to each cleft) appeared in a complementary manner, finally resulting in three repeated, intercalated rostral and caudal domains within the broad proximal pharyngeal ectoderm (Fig. 2C). Meanwhile, the dorsal-caudal neurogenic patches, which delaminate neurons to contribute to epibranchial ganglia, and the *Fgf3^+^* caudal patches were both differentiated from the caudal *Sox2^+^* pre-neural domains (Zhang et al., 2017). The specification of rostral domains served as segregations for the three discrete caudal domains, providing an intriguing morphological explanation for the metameric patterning of geniculate, petrosal and nodose placodes (Fig. 2C).

### Regionalized expression of Notch factors during epibranchial specification

**Fig. 3.**
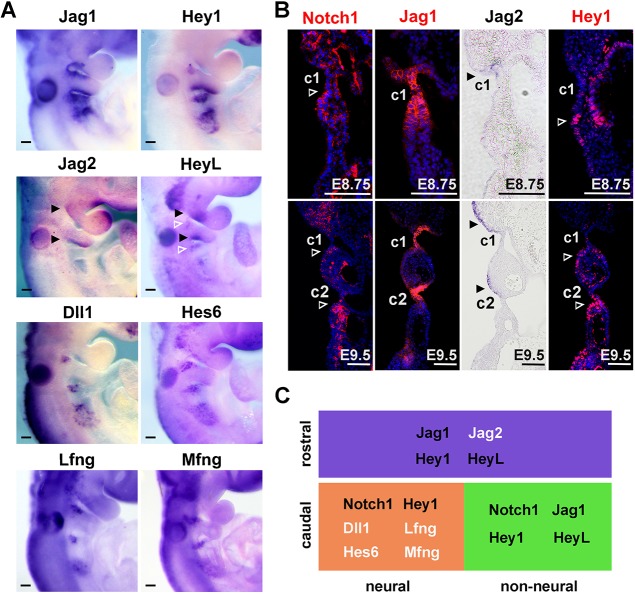
**Regionalized expression of Notch signalling factors during posterior placodal area specification.** (A) Whole-mount *in situ* hybridization showing *Jag1*, *Hey1*, *Jag2*, *HeyL*, *Dll1*, *Hes6*, *Lfng* and *Mfng* expression in WT embryos at E9.5 (*n*≥3 for each probe). (B) Coronal sections of WT embryos showing Notch1, Jag1, Hey1 and *Jag2* expression at E8.75 and E9.5. Scale bars: 100 µm. In A and B, black filled arrowheads indicate positive signals at domains rostral to the clefts; white open arrowheads indicate signals at domains caudal to the clefts. c1 and c2 indicate the first and second pharyngeal clefts. (C) Schematic showing regionalized expression of distinct Notch factors at the rostral, non-neural caudal and neural dorsal-caudal domains.

Notch signalling has been shown to regulate placodal cell differentiation in olfactory, otic and other placodes. In order to determine whether Notch signalling is involved during the individualization of epibranchial placodes from the broader PPA, the spatiotemporal expression patterns of Notch signalling factors were examined. The Notch ligand *Jag1* and the Notch target gene *Hey1* were confined to the c1 and c2 regions at E9.5 (Fig. 3A). *Jag1* was expressed early within the PPA region at around E8.5 (Fig. S2) and restricted to otic placode and cleft regions at E9.5 (Fig. 3B) (Zhang et al., 2017). *Hey1* expression could be detected from E8.75 and was later restricted to the caudal domains (Fig. 3B; Fig. S2). *HeyL* was detected in both the rostral and caudal domains (Fig. 3A). *Jag2* expression appeared at around E8.75 at the rostral region of c1 (Fig. 3B), and *Jag2* was expressed at the rostral regions of c1 and c2 at E9.5 (Fig. 3A,B), illustrating a rostral domain-restricted expression pattern. *Dll1* expression first appeared at both rostral and caudal regions at E9.0 (Fig. S2). However, the expression of *Dll1* was specifically restricted to the dorsal neurogenic regions at E9.5 (Fig. 3A). Another Hes family member, *Hes6*, was specifically expressed in the dorsal neurogenic domain (Fig. 3A). *Dll3* was not detected at the PPA at E9.5. *Hes1*, which has been reported to function in PA development but not in the epibranchial placode (Kameda et al., 2013; van Bueren et al., 2010), was contiguously expressed at the whole pharyngeal ectoderm (Fig. S2).

Among the three Fringe genes, which encode glycosyltransferases that modulate Notch receptors by glycosylation, *Rfng* was not expressed in the epibranchial territory, whereas *Mfng* was restricted to the dorsal-caudal neurogenic domain as well as migrating neuroblasts (Fig. 3A). *Lfng* was detected at the dorsal-caudal neurogenic domain, with weak expression at the ventral-caudal domains as well (Fig. 3A). We have previously reported the expression of Notch1 in the proximal pharyngeal ectoderm (Zhang et al., 2017). At E9.5, Notch1 was highly expressed at the caudal ectoderm relative to each cleft, and also in the mesenchyme (Fig. 3B), in line with a previous study (Williams et al., 1995).

In summary, these data show that the rostral and caudal domains are endowed with distinct expression patterns for genes in the Notch signalling pathway (summarized in Fig. 3C), suggesting a role for Notch signalling in the differentiation and segregation of the epibranchial placodes from the PPA.

### Notch signalling mediates the regional specification of proximal pharyngeal ectoderm

**Fig. 4.**
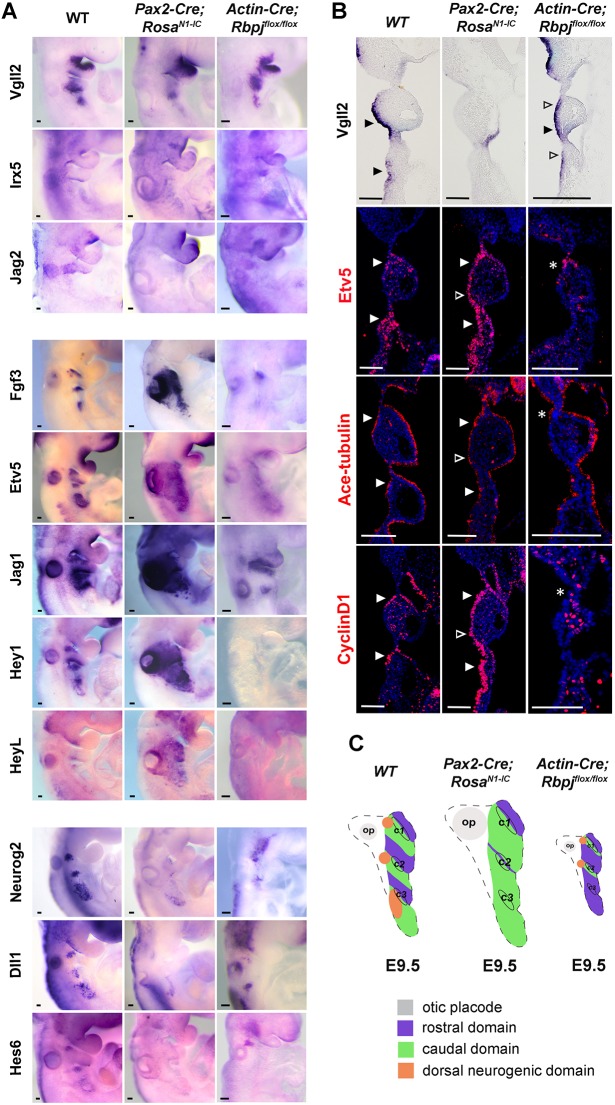
**Expression of epibranchial placodal genes in Notch signalling mutants.** (A) Whole-mount *in situ* hybridization showing *Vgll2*, *Irx5*, *Jag2*, *HeyL*, *Fgf3*, *Etv5*, *Jag1*, *Hey1*, *Neurog2*, *Dll1* and *Hes6* expression in WT, *Pax2-cre;Rosa^N1-IC^* and *Actin-cre;Rbpj^flox/flox^* embryos (*n*=3 for each genotype and probe) at E9.5. (B) Whole-mount *in situ* hybridization for *Vgll2* in coronal embryo sections, and immunostaining of Etv5, acetylated tubulin and cyclin D1 on coronal sections of embryos at E9.5 in the indicated genotypes (*n*=3 for each genotype and marker). Filled arrowheads represent normal expression domains, open arrowheads indicate ectopic expression domains. Asterisks indicate residual signals of indicated markers in pharyngeal ectoderm of *Actin-cre;Rbpj^flox/flox^* embryos. Scale bars: 100 µm. (C) Schematic summary of epibranchial placode rostral and caudal domain patterning phenotypes in gain-of-function and loss-of-function Notch mutant embryos at E9.5. c1, c2, c3 indicate the first, second and third pharyngeal clefts; op, otic placode.

To investigate the effect of Notch signalling on patterning the proximal pharyngeal ectoderm along the rostrocaudal and dorsoventral axes, the expression of regional markers was examined in *Pax2-Cre;Rosa^N1-IC^* embryos, in which N1ICD is activated in the PPA using the *Pax2-Cre* driver from E8.5 to E9.5, or in *Actin-Cre;Rbpj^flox/flox^* embryos, in which *Rbpj* was deleted by *Actin-Cre*, leading to a loss of the canonical Notch signalling activity from the zygotic stage. Whereas *Vgll2* was normally localized at the rostral domains in wild-type (WT) E9.5 embryos (Fig. 4A,B), expression of *Vgll2* was specifically lost in the pharyngeal ectoderm of *Pax2-Cre;Rosa^N1-IC^* embryos (Fig. 4B). Interestingly, in *Actin-Cre;Rbpj^flox/flox^* embryos expression of *Vgll2* expanded into the caudal domains of c1 and c2, covering the entire proximal PA ectoderm (Fig. 4A,B). The *Vgll2* expansion was not observed in the mandibular arch of the *Rbpj* mutant, in keeping with the presence of Notch signalling factors in the PPA but not in mandibular arch (Fig. 3). The effect of Notch signalling on caudal domain genes was the opposite of the effect on rostral domain genes. Expression of *Fgf3* was elevated when Notch activity was high, whereas the *Fgf3^+^* caudal domains were greatly reduced in the *Rbpj* mutant embryos (Fig. 4A). The expression of *Etv5*, which is one of the downstream targets of FGF signalling and as such an indicator of FGF activity, was upregulated in *Pax2-Cre;Rosa^N1-IC^* embryos but downregulated in *Actin-Cre;Rbpj^flox/flox^* embryos (Fig. 4A,B). This indicates that, although canonical Notch signalling was not required for the initiation of *Etv5* and *Fgf3* expression, it was essential for their maintenance, and that FGF activity was also under the regulation of Notch signalling. Moreover, the expression of *Neurog2*, one of the earliest markers for neural precursors in epibranchial placodes, was inhibited when N1ICD was activated (Fig. 4A), which is consistent with the function of Notch signalling in inhibiting neural differentiation in multiple neural systems. The expression of *Neurog2* was slightly reduced when canonical Notch activity was inhibited (Fig. 4A).

To investigate the distribution of Notch ligands and target genes that may be involved in the rostral-caudal patterning process, we examined the expression of *Jag1*, *Jag2*, *Dll1*, *HeyL*, *Hey1* and *Hes6* in response to activation or loss of Notch signalling. The expression of *HeyL* and *Hey1* was elevated when N1ICD was overexpressed in pharyngeal ectoderm and downregulated in *Actin-Cre;Rbpj^flox/flox^* embryos (Fig. 4A), suggesting that they are Notch targets in this context. In contrast, the expression of *Hes6*, as well as of *Neurog2*, was lost in *Pax2-Cre;Rosa^N1-IC^* embryos, and slightly reduced in *Actin-Cre;Rbpj ^flox/flox^* embryos (Fig. 4A). Notably, we found that the expression of *Jag2* and *Dll1* was inhibited, whereas the expression of *Jag1* was enhanced, by elevated Notch signalling (Fig. 4A).

To characterize the rostral and caudal cell fates in the Notch mutants, we examined the distribution of acetylated tubulin, which was highly expressed in caudal cells in WT embryos. When Notch was activated, acetylated tubulin was broadly expressed in the whole epibranchial territory (Fig. 4B). In contrast, inhibition of canonical Notch in the *Actin-Cre;Rbpj^flox/flox^* embryos led to decreased expression of acetylated tubulin in the pharyngeal ectoderm, whereas the endodermal expression remained similar to that observed in WT embryos (Fig. 4B). In summary, these observations demonstrate that the rostral *Vgll2* expression was inhibited whereas the caudal *Fgf3*, *Etv5* and acetylated tubulin expression was expanded when Notch activity was elevated (Fig. 4C). Conversely, a low level of canonical Notch activity through inhibition of *Rbpj* resulted in expansion of rostral markers into caudal domains, whereas the expression of caudal genes was significantly reduced (Fig. 4C).

### A cell-autonomous role of Notch signalling in regulating the rostrocaudal specification of proximal pharyngeal ectoderm

**Fig. 5.**
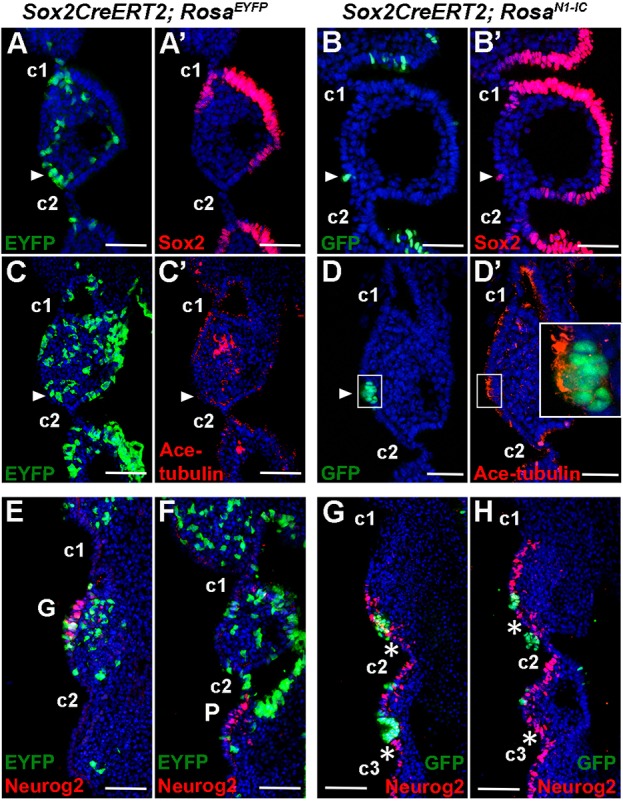
**Analysis of pharyngeal epithelial markers in Notch1 gain-of-function mutant showing cell fate changes in the rostral epithelial domain.** (A-H) Immunostaining of EYFP, GFP, Sox2, acetylated tubulin and Neurog2 on coronal sections of *Sox2creERT2;Rosa^EYFP^* and *Sox2creERT2;Rosa^N1-IC^* (N1ICD is linked with IRES-GFP) embryos at E9.5 (*n*=3 for each genotype and markers). Scale bars: 100 µm. Tamoxifen was injected at E7.5, and the embryos examined at E9.5. Sox2-expressing caudal-like cells (B′) and Neurog2^+^ pre-neural cells (G,H) were found in domains rostral to the clefts when Notch signalling is activated. Arrowheads indicate EYFP^+^ or GFP^+^ (N1ICD^+^) cells in rostral domains; asterisks indicate ectopic Neurog2^+^ cells in domains rostral to the clefts. Inset in D′ shows higher magnification of the boxed area. c1, c2, c3 indicate the first, second and third pharyngeal clefts; G, geniculate placode; P, petrosal placode.

We next addressed whether Notch signalling exerted a cell-autonomous role in specifying the proximal pharyngeal ectoderm. To this end, we used *Sox2CreERT2* activation to label epithelial cell clones in a mosaic fashion. By tamoxifen induction at E7.5 and harvesting the *Sox2CreERT2;Rosa^EYFP^* embryos at E9.5, EYFP^+^ cells could be found in both rostral and caudal domains (Fig. 5A,C). This suggests that both the *Sox2*-negative rostral and the *Sox2*-positive caudal cells originated from early *Sox2^+^* progenitors, consistent with our results shown in Fig. 1 using *Pax2-Cre*. We activated N1ICD in the PPA cells of *Sox2creERT2*;*Rosa^N1-IC^* embryos by tamoxifen induction at E7.5, and cells with induced N1ICD expression would be identified by expression of GFP which was linked to N1ICD via an IRES element. Some GFP^+^ cells were found at the rostral domains in E9.5 *Sox2creERT2;Rosa^N1-IC^* embryos (Fig. 5B,D). Strikingly, these GFP^+^ cells were highly *Sox2*^+^ (Fig. 5B′) and expressed high levels of acetylated tubulin (Fig. 5D′), indicating a caudal cell identity. Expression of Sox2 and acetylated tubulin was confined to the rostral cells exhibiting GFP (and by inference N1ICD) activity but was not observed in surrounding GFP-negative cells, indicating a cell-autonomous role of Notch signalling in activating and/or maintaining the *Sox2* expression and multi-ciliated morphology. However, although the effect on *Sox2* and acetylated tubulin expression was confined to the N1ICD-GFP*^+^* cells in the rostral domain, we observed that cells immediately adjacent to the N1ICD-GFP*^+^* cells were *Neurog2^+^* (Fig. 5E-H). Collectively, these observations indicate that high Notch activity in rostrally located cells can induce a caudal fate switch, and that adjacent cells as a consequence take on *Neurog2* expression.

## DISCUSSION

**Fig. 6.**
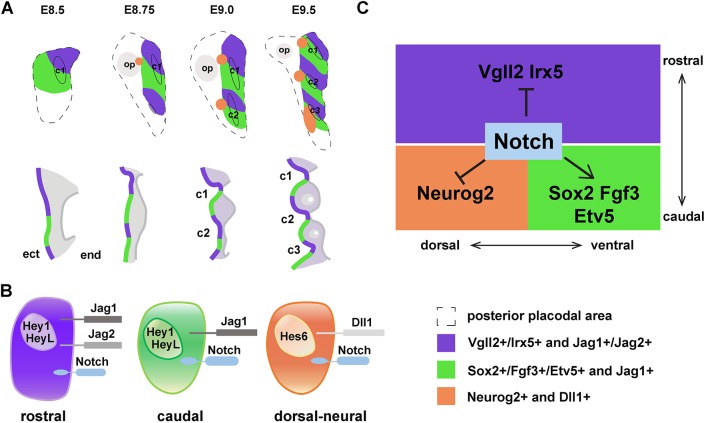
**Model for how Notch signalling may regulate early rostral-caudal and dorsal-ventral regionalization of the epibranchial placode and proximal pharyngeal ectoderm.** (A) Stepwise segregation of posterior placodal area into geniculate, petrosal and nodose placodes (orange) as well as rostral (purple) and caudal (green) epibranchial epithelial domains from E8.5 to E9.5. The upper panel illustrates the sagittal view, while the lower panel depicts the coronal section view. Black oval lines indicate positions of pharyngeal clefts; dashed lines demarcate the posterior placodal area. c1, c2, c3 indicate the first, second and third pharyngeal clefts; op, otic placode. (B) Schematic illustrating the expression of Notch signalling factors in the rostral, caudal and dorsal- neural epibranchial placodal cells. (C) Proposed model for the role of Notch signalling in regulating regionalization and cell fate specification of the proximal pharyngeal ectodermal cells.

This study lays out the process of stepwise individualization of epibranchial placodes from the broader *Pax2*^+^ PPA. First, the *Pax2*-expressing PPA gave rise to not only otic and epibranchial placodes, but also to proximal pharyngeal surface ectoderm. Second, the epibranchial territory of the PPA encompassed the proximal pharyngeal ectoderm and expanded caudally from the first pharyngeal arch as the second and third pharyngeal arches were generated. Subsequently, within the broad *Eya1^+^/Six1^+^* region, accompanying the appearance of each pharyngeal cleft, a *Vgll2^+^/Irx5^+^* domain and a *Sox2^+^/Fgf3^+^/Etv5^+^* domain appeared at the rostral and caudal positions of each cleft, respectively. The sequential patterning resulted in intercalated rostral and caudal repeating domains along the epibranchial region. In parallel, the *Neurog2^+^* neurogenic patches were induced at the dorsal edge of each caudal domain and gradually downregulated *Sox2* expression, whereas the ventral portions remained as *Sox2^+^/Fgf3^+^* (Zhang et al., 2017). Ultimately, the regionalization of the proximal pharyngeal ectoderm in a rostrocaudal sequence led to the appearance of three discrete *Neurog2^+^* epibranchial placodes (summarized in Fig. 6). Notch signalling contributed to the subdivision of *Pax2^+^* PPA both rostrocaudally and dorsoventrally: high levels of Notch activity led to expanded expression of caudal genes into rostral domains and inhibited neural differentiation in the dorsal-caudal placodal region.

### Regional specification and patterning of epibranchial placodes

Lineage tracing by *Pax2-Cre* and *Sox2-CreERT2* showed that epibranchial precursors gave rise to a wide range of derivatives, including not only epibranchial neurons, but also the rostral and caudal proximal pharyngeal ectodermal cells, supporting an origin from the placodal region for these ectodermal derivatives. Lineage tracing by *Sox2-CreERT2;Rosa^EYFP^* with tamoxifen injected at E7.5 and E8.5 revealed that both the rostral and caudal cells were labelled by EYFP at E9.5. In contrast, the caudal cells were Sox2 negative, suggesting that the rostral non-neural cells lost the Sox2 expression at around E9.5 (Fig. 1; Fig. S1). Moreover, the rostral *Vgll2^+^/Irx5^+^* cells showed a thin and cuboidal morphology versus a thickened ciliated morphology for the ventral-caudal *Sox2^+^/Fgf3^+^/Etv5^+^* cells (Fig. 1), further underscoring their distinct fates.

Our data provided evidence for regional patterning of epibranchial placodes, through which the placodal (caudal) cells and the interplacodal (rostral) cells were specified into distinct fates with different transcriptional profiles and cellular morphology. In line with this notion, the interplacodal cells exhibited a loss of placodal signature revealed by reduced *Sox2* and *Pax2* expression, accompanied by a local gain of *Vgll2* expression (Fig. 2). Furthermore, interplacodal and placodal cells became morphologically different (Fig. 1). This is in agreement with a previous observation that the entire branchial region is initially covered with thick ectoderm that becomes thinner from around E8.75 (10 ss), leaving thicker cells in the epibranchial placodes (Baker and Bronner-Fraser, 2001; Verwoerd and van Oostrom, 1979). Our observations are also consistent with previous studies in chick, mouse and human embryos, in which the epibranchial placodes arose from more extended areas of thickened branchial ectoderm (Abu-Elmagd et al., 2001; Baker and Bronner-Fraser, 2000; D'Amico-Martel and Noden, 1983; Müller and O'Rahilly, 1988; Washausen and Knabe, 2013, 2017; Washausen et al., 2005). Moreover, our data corroborate a previous report of thickened morphology in the posterior placodal area in the Northern treeshrew (*Tupaia belangeri*) (Washausen et al., 2005).

### The cellular mechanism for the individualization of epibranchial placodes

How the geniculate, petrosal and nodose placodes are physically segregated from each other is a long-standing question. Two possible scenarios include local sorting of intermingled pre-defined progenitors or regional cell fate changes of multipotent precursors in response to exposure to regional signals (Breau and Schneider-Maunoury, 2014; McCabe and Bronner-Fraser, 2009). The results presented here support a scheme in which multipotent precursors are regionally specified into epibranchial placodal cells as well as non-placodal cells. In line with this, before the individual epibranchial placodes become molecularly distinct from the surrounding ectoderm, *Eya1*, *Six1* and *Sox2* were expressed in the broad contiguous proximal pharyngeal ectoderm (Fig. 2). Shortly thereafter, the expression of Sox2 began to become progressively restricted to smaller subdomains located at the caudal side of each cleft, whereas the expression of *Vgll2* began to appear in complementary rostral subdomains, thereby subdividing the broad proximal pharyngeal ectoderm by establishing distinct *Sox2^+^* and *Vgll2^+^* regions. *Neurog2* expression was induced at the dorsal edge of the *Sox2^+^* caudal subdomains, making epibranchial placodes molecularly distinct from other proximal pharyngeal ectodermal cells. Notably, from E8.5 onwards, the expression of *Sox2* and *Vgll2* was likely to be mutually exclusive, and *Sox2^+^* and *Vgll2^+^* cells were morphologically distinct, suggesting that the two different cell fates are regionally specified, rather than sorted from intermingled precursors. Moreover, lineage-tracing analysis using *Sox2CreERT2;Rosa^EYFP^* revealed that both rostral and caudal cells originated from *Sox2^+^* progenitors, further supporting regional specification from a common pool of *Sox2^+^* progenitors.

### Patterning of the proximal pharyngeal epithelia and pharyngeal arch development

Previous studies of pharyngeal patterning focused on the anteroposterior organization of the ‘Hox code’, which provided identities for the PAs, except for the Hox-free PA1 (Grammatopoulos et al., 2000). For each PA, the proximodistal organization was characterized by the ‘Dlx code’ (Minoux and Rijli, 2010). In this study, we showed that in the proximal pharyngeal region, the surface epithelium was not homogeneous, but distinctly patterned along the rostrocaudal axis. As such, for each proximal PA, the anterior half was covered with *Sox2^+^/Fgf3^+^/Etv5^+^* ectodermal cells, whereas the posterior half was *Vgll2^+^/Irx5^+^*. Notably, the rostrocaudal patterning of placodal epithelial cells was observed from the region rostral to the first pharyngeal cleft but the rest of the mandibular arch was not included. Furthermore, the specification of rostrocaudal epithelial identity and the process of pharyngeal segmentation were temporally coordinated. It was previously suggested that an additional physical boundary was required to ensure the stable segregation of cells with distinct identities (Schlosser, 2006), and the formation of pharyngeal clefts, which fused with pharyngeal pouches to form pharyngeal segments, may serve as such a boundary between the rostral and caudal domains of the proximal pharyngeal surface ectoderm. It is possible that the rostrocaudal epithelial specification might instruct the segmentation process, and loss of caudal domain factors, such as *Sox3* and FGF signalling in mice does indeed lead to defective segmentation (Rizzoti and Lovell-Badge, 2007; Trokovic et al., 2003). Once in contact with the pharyngeal cleft, the pharyngeal pouch would produce a BMP signal to further induce neurogenesis in each caudal domain (Begbie et al., 1999).

### The role of Notch in specifying rostrocaudal and dorsoventral regionalization of the proximal pharyngeal ectoderm

Our data point to an important role for Notch signalling in controlling the development of the rostral and caudal domains. Notch activation enhanced the differentiation of *Pax2^+^* precursors into *Sox2^+^/Fgf3^+^/Etv5^+^* caudal cells, at the expense of *Vgll2^+^/Irx5^+^* rostral cells and *Neurog2^+^* dorsal-caudal pre-neural cells. Conversely, inhibition of canonical Notch signalling by deletion of *Rbpj* led to differentiation of *Pax2^+^* precursors to *Vgll2^+^/Irx5^+^* rostral cells instead of to *Sox2^+^/Fgf3^+^/Etv5^+^* caudal cells. In the *Rbpj* knockout mutant embryos, expression of *Neurog2* was reduced (Fig. 4A), which is consistent with the requirement of Notch signalling for induction of the pre-neural fate (de la Pompa et al., 1997). In line with promotion of a caudal fate, high Notch signalling favoured differentiation of acetylated tubulin^+^ ciliated cells at the expense of the flattened cells and neurons. This provides evidence for a role of Notch signalling in the early phases of epibranchial placode differentiation and extends previous reports on a role for *Dll1* in generation of petrosal and nodose ganglia (Begbie et al., 2002; de la Pompa et al., 1997) and in segregation of neuronal and non-neuronal cells in the epibranchial placodes (Zhang et al., 2017).

The mosaic expression of N1ICD in the *Sox2-CreERT2* embryos, where N1ICD-GFP*-*expressing cells were *Sox2^+^* and acetylated tubulin^+^, revealed a cell-autonomous role for N1ICD. Interestingly, cells adjacent to the N1ICD-GFP-expressing cells exhibited an altered fate, with elevated *Neurog2* expression, which likely is a secondary effect of the changed fate in the N1ICD-GFP-expressing cells. This constitutes a classical example of lateral inhibition or specification, a mechanism ensuring segregation of distinct fates in a field of initially homogeneous cells. Lateral inhibition regulated by Notch signalling has been extensively demonstrated in *Drosophila* and in a few situations in mammals, such as inner ear development (for a review, see Sjöqvist and Andersson, 2019).

Genes in the Notch signalling pathway showed complex expression patterns during epibranchial differentiation and in the rostral and caudal domains, notably with regard to Notch ligand and Fringe gene expression (summarized in Fig. 3C). The subdivision into a *Dll1*- and Fringe-expressing territory versus a *Jag1*-expressing territory is very similar to what is observed during dorsoventral patterning and specification of the developing spinal cord (Marklund et al., 2010), and likely regulates how Notch signalling is deployed in segregating and maintaining the identities of the rostral and dorsal domains. Modification of Notch receptors by Fringe proteins enhances Dll1-Notch signalling, while repressing Jag1-Notch signalling (Harvey and Haltiwanger, 2018; Siebel and Lendahl, 2017). As for the developing spinal cord (Marklund et al., 2010), this may lead to intra-domain Notch signalling in the caudal neural domain, while signalling is abrogated across the boundary to the rostral domain. The *Dll1/*Fringe and *Jag1* domain-specific expression patterns observed here are in contrast to, for example, the situation in the *Drosophila* wing blade, where the presence of *Ser/Fng*- and *Dll1*-expressing domains ensure inter-domain Notch activation only at the boundary (in the wing blade), but not within the different domains (Wu and Rao, 1999).

How Notch signalling controls the upregulation of certain genes (*Sox2*, *Fgf3* and *Etv5*) while repressing other genes (*Neurog2*, *Vgll2* and *Irx5*) (summarized in Fig. 6C) is not fully understood. Repression of gene activity may involve Notch downstream genes of the Hes and Hey family, whereby *Hey1* is expressed in all domains, whereas *Hes6* expression is confined to the caudal neural domain. Hes genes have been shown to antagonize expression of proneural genes (Ohtsuka et al., 1999). There are indeed a number of potential Hes-binding sites in or near the *Vgll2* and *Irx5* genes, but it has not yet been experimentally established whether Hes proteins dynamically bind to these sites. Upregulation may be a direct consequence of activation via the NICD/MAML/CSL ternary complex, but in chromatin immunoprecipitation experiments in cell lines CSL binding to potential binding sites near the *Sox2* and *Fgf3* genes was not observed, indicating that the effect may be indirect. In conclusion, this study sheds new light onto early epibranchial specification by providing evidence for a non-neural developmental trajectory from the epibranchial precursors and a role for Notch in this process.

## MATERIALS AND METHODS

### Experimental animals

The mouse lines used in this study include wild-type C57BL/6N, *Rosa^N1-IC^* (Murtaugh et al., 2003); *Rbpj^flox/flox^* (Han et al., 2002); *Rosa^EYFP^* (Srinivas et al., 2001); *Rosa^l^^acZ^* (Soriano, 1999); *Irx5^EGFP/+^* (Li et al., 2014); *Pax2-cre* (Xu et al., 2002) and *Sox2-creERT2* (Arnold et al., 2011). Mice were maintained on a C57BL/6N background and housed at the Laboratory Animal Unit at the University of Hong Kong. Genotyping was conducted by PCR using the primers listed in Table S1. To activate Cre activity in *Sox2creERT2* embryos, tamoxifen (Sigma-Aldrich, 06734) was dissolved in corn oil (20 mg/ml) and administered by intraperitoneal injection to pregnant females (0.1 mg/g body weight) at E7.5 to E9.5. For each stage and each genotype, at least three embryos were collected for further analysis. All mouse experiments were approved by the University of Hong Kong Committee for Use of Live Animals for Teaching and Research (CULATR No. 4357-17).

### Riboprobe labelling

Plasmids with target cDNA sequences were obtained from other laboratories or cloned in this study as shown in Table S2. The cDNA plasmids were linearized with restriction enzymes (summarized in Table S3), separated on 1% low melting agarose gel and purified. DNA samples were dissolved in DEPC H_2_O; 1 μg purified DNA was used for reverse transcription and digoxigenin labelling (Sigma-Aldrich, 11093274910). Labelled RNA probes were precipitated in ethanol, air-dried, dissolved in DEPC H_2_O and stored at −80°C.

### Whole-mount X-gal staining and *in situ* hybridization

The activity of β-galactosidase in *Rosa^lacZ^* reporter was analysed by X-gal staining as described by Kwan et al. (2001). Briefly, embryos were fixed in 0.2% glutaraldehyde, 1.5% formaldehyde at 4°C for 30-90 min. The embryos were then washed and stained in the dark in 1 mg/ml X-gal (Sigma-Aldrich, 7240906) at room temperature for 1 h.

For *in situ* hybridization, embryos from E8.5 to E9.5 were harvested in cold DEPC PBS and fixed in 4% paraformaldehyde at 4°C overnight. Embryos were then dehydrated with a graded series of DEPC methanol/PBST (PBS with Tween-20) solution, and stored at −20°C or used immediately. Before performing *in situ* hybridization, the embryos were rehydrated, treated with protease K (Sigma-Aldrich, P8811) and then post-fixed with 4% paraformaldehyde, 0.2% glutaraldehyde and washed in PBST. Embryos were pre-hybridized by incubating at 55-65°C for 3 h; digoxigenin-labelled riboprobes were then added into the hybridization mix and incubated at 55-65°C overnight. After hybridization, embryos were washed in hybridization mix/MABT (maleic acid buffer with Tween-20) solution, then blocked with 10% blocking reagent and 20% heat-inactivated horse serum. Anti-digoxigenin-alkaline phosphatase (Sigma-Aldrich, 11093274910) was added to the embryos and incubated overnight at 4°C. After washing for 24 h, embryos were immersed in BM Purple substrate (Roche) until a clear signal was detected. The reaction was stopped by washing in PBST followed by post-fixation with 4% paraformaldehyde.

### Histology and immunohistochemistry

Mouse embryos were fixed in 4% paraformaldehyde at 4°C for 1 h or overnight, according to the requirement of specific antibodies. After fixation, embryos were washed in PBS, incubated in 15% sucrose overnight at 4°C, then transferred to gelatin solution and incubated at 37°C. Embryos were embedded in gelatin block, cut to a suitable size and stored at −80°C before 10-μm-thick sections were prepared.

For immunostaining, histological sections were blocked with 10% horse serum in PBS at room temperature for 1 h; primary antibodies were applied to the sections and incubated at 4°C overnight. Sections were then washed and incubated with fluorescence-linked secondary antibodies for 1 h at room temperature. At this step, DAPI (Sigma-Aldrich, D9542) was co-stained with secondary antibodies. Histological slides were then washed in PBS before being mounted with mounting medium (Vectashield, Vector Laboratories). Images were captured using an Olympus fluorescence microscope (BX51). At least three different sections from three different embryos were stained with each primary antibody. The primary and secondary antibodies used are summarized in Table S4.

### TUNEL assay

Histological sections were incubated in permeabilization solution (0.1% Triton X-100, 0.1% sodium citrate) for 2 min on ice, then washed twice with PBS. Slides were incubated with TUNEL reaction mixture (Roche, 1684795910; enzyme solution and label solution at 1:9 ratio) at 37°C for 30 min, followed by three washes in PBS. Sections were mounted in mounting medium (Vectashield).

## Supplementary Material

Supplementary information

Reviewer comments
