# Supplementary material for: Notch signalling regulates epibranchial placode patterning and segregation
Source: Development. 2020 Feb 17;147(4):dev183665. doi: 10.1242/dev.183665 (PMC7044445; doi:10.1242/dev.183665)
Supplement: Supplementary information [file develop-147-183665-s1.pdf]

Figure S1

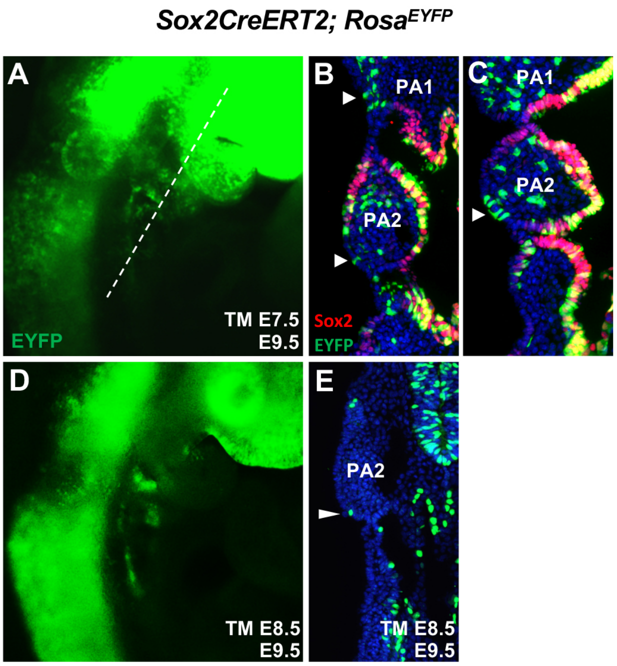

Figure S1. Lineage tracing of the Sox2+ placodal precursors in *Sox2CreERT2; Rosa^{EYFP}* embryos at E9.5. (A and D) Whole mount *EYFP* fluorescence at E9.5 after the tamoxifen (TM) injection at E7.5 (A) (n=4) and E8.5 (D) (n=2). (B, C and E) Coronal section of the E9.5 *Sox2CreERT2; Rosa^{EYFP}* embryos with tamoxifen injected at E7.5 (B and C) (n=4) and at E8.5 (E) (N=2). Arrows indicate the EYFP+ cells in pharyngeal ectoderm. PA, pharyngeal arch.

Development • Supplementary information

Figure S2

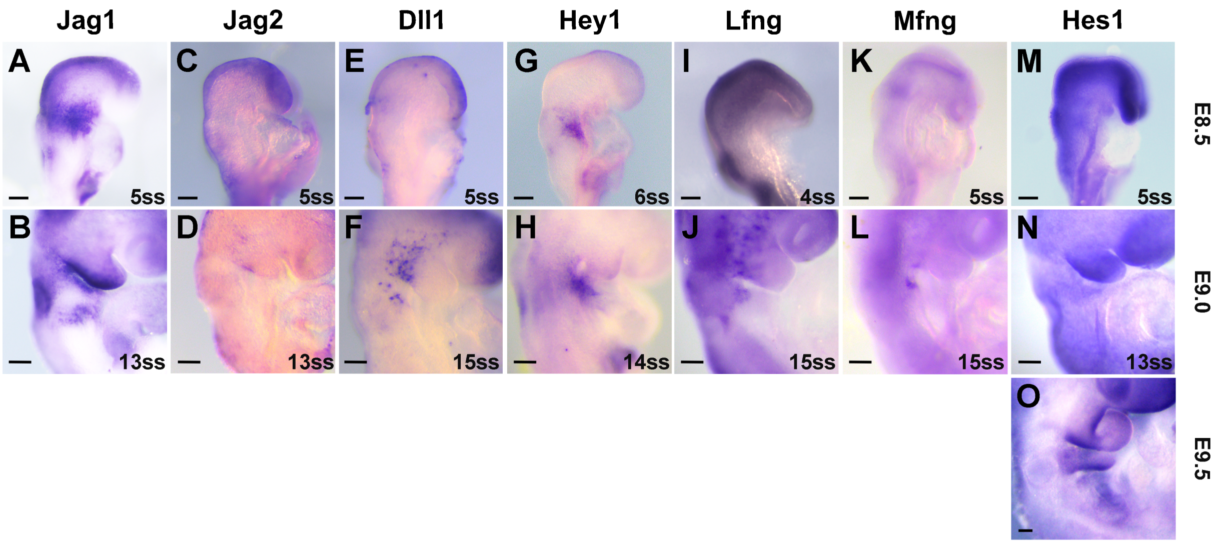

Figure S2. Distinct expression of Notch signalling factors during the early stage of posterior placodal area specification. (A-O) Whole mount In situ hybridization showing *Jag1* (A and B), *Jag2* (C and D), *Dll1* (E and F), *Hey1* (G and H), *Lfng* (I and J), *Mfng* (K and L) and *Hes1* (M-O) expression on WT embryos at indicated stages (n≥3 each stage).

Figure S3

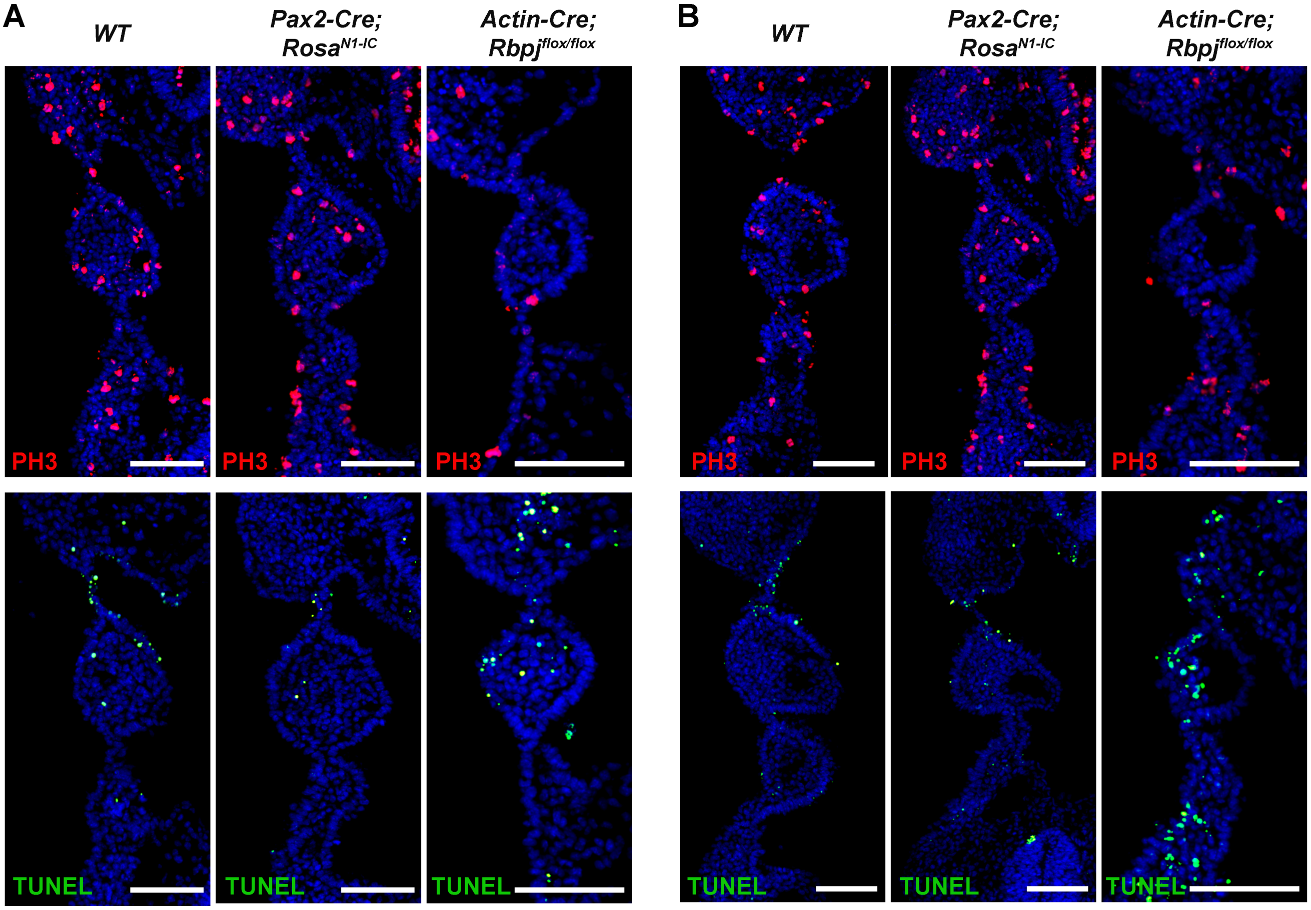

Figure S3. Cell apoptosis and proliferation analysis on *WT*, *Pax2-Cre;Rosa$^{N1\text{-}IC}$* and *ActinCre;Rbpj$^{flox/flox}$* embryos. (A and B) Immunostaining of phospho-histone H3 (PH3) and TUNEL on coronal sections of *WT*, *Pax2-Cre;Rosa$^{N1\text{-}IC}$* and *Actin-Cre;Rbpj$^{flox/flox}$* embryos at E9.0 (A) (n≥2) and E9.5 (B) (n=3). Scale bar = 100 μm.

Development • Supplementary information

Supplementary Tables

Table S1: Sequences of primers for genotyping of mouse lines

Mouse line	Primer	Sequence
Cre	Forward (F)	5' ACGGAAATCCATCGCTCGACCAGTT 3'
	Reverse (R)	5' GTCCGGGCTGCCACGACCAA 3'
$Irx5^{+}$	F	5' GGTCCCGAAGGGCCAGAATCAGAATTGGGG 3'
	R	5' GCATTCTTCCGGTACGCGGGGTCCCCATA 3'
$Irx5^{EGFP}$	F	5' GGTCCCGAAGGGCCAGAATCAGAATTGGGG 3'
	R	5' CCGGTGGATGTGGAATGTGTGCGAGGCCA 3'
$RBPJ^{+}$	F	5' GTTCTTAACCTGTTGGTCGGAACC 3'
	R	5' GCTTGAGGCTTGATGTTCTGTATTGC 3'
$RBPJ^{flox}$	F	5' GTTCTTAACCTGTTGGTCGGAACC 3'
	R	5' GCAATCCATCTTGTTCAATGGCC 3'
$RBPJ^{-}$	F	5' GCTTGAGGCTTGATGTTCTGTATTGC 3'
	R	5' CTGAGTAAGATGAGATGCTGACATCTGA 3'
$Rosa^{N1\text{-}IC}$	F	5' ACCCTGGACTACTGCGCCC 3'
	R	5' CGAAGAGTTTGTCCTCAACCG 3'

Table S2: Sequences of primers for cDNA cloning

cDNA	Forward	Reverse
Dll1	5' ATCTGTCTGCCAGGG 3'	5' GCACCGTTAGAACAA 3'
Hes6	5' AGTAGTTTGCACTAG 3'	5' AGAACCTCGGCGTTC 3'
Hey1	5' ATGAAGAGAGCTCAC 3'	5' TTAGAAAGCTCCGAT 3'
HeyL	5' AGTATTGGGTTTCGG 3'	5' TGATTTCTGAGACCC 3'
Mfng	5' AGCTGGTGCGGTTCT 3'	5' ATCCCCTCCCACACA 3'
Vgll2	5' TCCTCATTTTCCAAC 3'	5' TAGGCAGAGGCTTGT 3'

Table S3: Probes for in situ hybridization

Probes	Restriction enzyme for anti-sense probe	Polymerase for transcription	Reference
Dll1	BamHI	T7	this study
Eya1	SalI	T7	Xu et al., 1997
Etv5	HindIII	T3	Kindly provided by Frank Costantini
Fgf3	SalI	T7	Wilkinson et al., 1988
Hes6	BamHI	T7	this study
Hes1	EcoRI	T7	Zheng et al., 2000
Hey1	BamHI	T7	this study
HeyL	BamHI	T7	this study
Jag1	EcoRI	T3	Mitsiadis et al., 1997
Lfng	HindIII	T7	Zhang et al., 1998

Mfng	BamHI	T7	this study
Neurog2	BamHI	T7	Gradwohl et al., 1996
Notch1	Apa1	SP6	Williams et al., 1995b
Six1	BamHI	T3	Oliver et al., 1995
Sox2	AccI	T3	Avilion et al., 2003
Vgll2	BamHI	T7	this study

Table S4: Antibodies used for immunohistochemistry

Antibodies	Source	Identifiers	Dilutions
Donkey anti-Rabbit IgG (H+L) Highly Cross-Adsorbed Secondary Antibody, Alexa Fluor 488	Thermo Fisher Scientific	A21206, RRID:AB_2535792	1:500
Goat polyclonal anti-Notch1 (C20)	Santa Cruz Biotechnology	sc-6014, RRID: AB_650336	1:400
Goat polyclonal anti-Sox2	Neuromics	GT15098-100, RRID:AB_21955800	1:500
Mouse monoclonal anti-Acetylated-tubulin	Sigma-Aldrich	T7451, RRID:AB_609894	1:700
Mouse monoclonal anti-Neurog2	R&D systems	MAB3314, RRID:AB_2149520	1:1000
Mouse polyclonal anti-Islet1	DSHB	PCRP-ISL1-1A9, RRID:AB_2618775	1:400
Rabbit polyclonal anti-GFP	Abcam	ab6556, RRID:AB_305564	1:1000
Rabbit polyclonal anti-Hey1	Abcam	AB22614, RRID:AB_447195	1:500
Rabbit polyclonal anti-Pax2	Invitrogen	71-6000, RRID:AB_2533990	1:500
Rabbit polyclonal anti-Six1	Sigma-Aldrich	HPA001893, RRID:AB_1079991	1:500
Rat monoclonal anti-Jagged1	DSHB	Ts1.15h, RRID: AB_528317	1:300
Rabbit monoclonal anti-CyclinD1	Abcam	ab16663 RRID: AB_443423	1:300
Rabbit polyclonal anti-phospho-Histone H3 (Ser10)	Upstate	06-570 RRID: AB_310177	1:500
Sheep polyclonal anti-Digoxigenin-alkaline phosphatase	Sigma-Aldrich	Cat#11093274910	1:2000
In Situ Cell Death Detection Kit, Fluorescein	Roche	Cat#11684795910	NA

Development • Supplementary information

Supplementary References

Avilion, A. A., Nicolis, S. K., Pevny, L. H., Perez, L., Vivian, N. and Lovell-Badge, R. (2003). Multipotent cell lineages in early mouse development depend on SOX2 function. *Genes & development* **17**, 126-140.

Gradwohl, G., Fode, C. and Guillemot, F. (1996). Restricted expression of a novel murineatonal-related bHLH protein in undifferentiated neural precursors. *Developmental biology* **180**, 227-241.

Mitsiadis, T. A., Henrique, D., Thesleff, I. and Lendahl, U. (1997). Mouse Serrate-1 (Jagged-1): expression in the developing tooth is regulated by epithelial-mesenchymal interactions and fibroblast growth factor-4. *Development* **124**, 1473-1483.

Oliver, G., Wehr, R., Jenkins, N. A., Copeland, N. G., Cheyette, B. N., Hartenstein, V., Zipursky, S. L. and Gruss, P. (1995). Homeobox genes and connective tissue patterning. *Development* **121**, 693-705.

Wilkinson, D. G., Peters, G., Dickson, C. and McMahon, A. P. (1988). Expression of the FGF-related proto-oncogene int-2 during gastrulation and neurulation in the mouse. *The EMBO journal* **7**, 691-695.

Williams, R., Lendahl, U. and Lardelli, M. (1995). Complementary and combinatorial patterns of Notch gene family expression during early mouse development. *Mechanisms of development* **53**, 357-368.

Xu, P.-X., Woo, I., Her, H., Beier, D. R. and Maas, R. L. (1997). Mouse Eya homologues of the Drosophila eyes absent gene require Pax6 for expression in lens and nasal placode. *Development* **124**, 219-231.

Zhang, N. and Gridley, T. (1998). Defects in somite formation in lunatic fringe-deficient mice. *Nature* **394**, 374.

Zheng, J. L., Shou, J., Guillemot, F., Kageyama, R. and Gao, W.-Q. (2000). Hes1 is a negative regulator of inner ear hair cell differentiation. *Development* **127**, 4551-4560.

Development • Supplementary information
