# Supplementary material for: Notch signalling regulates epibranchial placode patterning and segregation
Source: Development. 2020 Feb 17;147(4):dev183665. doi: 10.1242/dev.183665 (PMC7044445; doi:10.1242/dev.183665)
Supplement: Reviewer comments [file develop-147-183665_review_history.pdf]

Notch signalling regulates epibranchial placode patterning and segregation

Li Wang, Junjie Xie, Haoran Zhang, Long Hin Tsang, Sze Lan Tsang, Eike-Benjamin Braune, Urban Lendahl and Mai Har Sham
DOI: 10.1242/dev.183665

Editor: Francois Guillemot

Review timeline
Original submission: 16 August 2019
Editorial decision: 11 September 2019
First revision received: 24 December 2019
Accepted: 14 January 2020

Original submission

First decision letter

MS ID#: DEVELOP/2019/183665

MS TITLE: Notch signaling regulates epibranchial placode patterning, segregation and differentiation

AUTHORS: Li Wang, Junjie Xie, Haoran Zhang, Long Hin Tsang, Sze Lan Tsang, Urban Lendahl, and Mai Har Sham

I have now received the reports of three referees on your manuscript and I have reached a decision. The reports are appended below and you can access them online: please go to BenchPressand click on the 'Manuscripts with Decisions' queue in the Author Area.

As you will see, all the referees express great interest in your work, but they also have some significant criticisms and recommend a substantial revision of your manuscript before we can consider publication. If you are able to revise the manuscript along the lines suggested, which may involve further experiments, I will be happy to receive a revised version of the manuscript. The identification of the Notch targets mentioned by referee 2 would be of course interesting but seems beyond the scope of the study unless you already have some evidence in that direction. The request of referee 3 that you analyse the effect of manipulations of Notch signalling at other stages than E9.5 will also require a significant amount of work without being necessarily informative. Instead, during the post-review discussion, referees asked that youdefine the time point when each Cre activates or inhibits Notch in the epibranchial territory and interpret the phenotype accordingly, i.e. if there is only a short time between Notch modulation and phenotype analysis, one could argue for an immediate effect. I agree this would be a valuable addition to your study.

Your revised paper will be re-reviewed by the original referees, and its acceptance will depend on your addressing satisfactorily all their major concerns. Please also note that Development will normally permit only one round of major revision. Please attend to all of the reviewers' comments and ensure that you clearly highlight all changes made in the revised manuscript. Please avoid using 'Tracked changes' in Word files as these are lost in PDF conversion. I should be grateful if you would also provide a point-by-point response detailing how you have dealt with the points raised by the reviewers in the 'Response to Reviewers' box. If you do not agree with any of their criticisms or suggestions please explain clearly why this is so.

© 2020. Published by The Company of Biologists under the terms of the Creative Commons Attribution License (https://creativecommons.org/licenses/by/4.0/).

Reviewer 1

Advance summary and potential significance to field

This study addresses how the epibranchial placodes, which contribute viscerosensory neurons to the cranial PNS, are patterned and segregate from the other during early mouse embryonic development. The authors first establish that the Pax2 expressing posterior placodal area (PPA) gives rise to the otic placode, epibranchial placodes as well as the epidermal regions in between and that the discrete epibranchial placodes form by the thinning out of these intervening epidermal domains. They then show that the regions of the PPA rostral and caudal to the pharyngeal clefts express different marker genes including several ligands and receptors of the Notch pathway. Finally, the authors use Notch gain and loss of function mouse mutants to show that Notch signaling plays an important role in the patterning of the PPA (with high Notch promoting rostral-to-cleft identity and low Notch promoting caudal-to-cleft identity).
The experiments described in this paper are straightforward and the results obtained overall well documented (see detailed comments below). The study makes an important contribution to our understanding of early ectodermal patterning in the vertebrate embryo. It is, therefore, of wide interest and suitable for publication in Development after some relatively minor concerns are addressed.

Comments for the author

Detailed comments:
- 128: here the authors need to make clearer that their use of “rostral” and “caudal” throughout the paper is meant in relation to the pharyngeal clefts (it is mentioned in passing but should be stressed more). I was initially confused and would have found it more appropriate to talk about the rostral and caudal part of each pharyngeal arch (in which case the “caudal” domains of the authors become “rostral”) since this is a more obvious anatomical unit. In the figure legends it would also be useful to explicitly write “rostral to the cleft” etc to avoid confusion. Also, the pharyngeal clefts are not always well visible in all figures. I suggest to mark the pharyngeal clefts clearly in all figs (that would be clearer than marking rostral and caudal expression domains with different arrowheads)
- 285: “Vgll2 expanded into the caudal domains”. This should be described in a bit more detail (1) in emphasizing that this is a rostral expansion into the ectoderm located caudal to a more anterior cleft) and (2) in explicitly describing, which pharyngeal arches are affected. It is notable that this is not observed for the mandibular arch: i.e. the rostral domain of the first pharyngeal pouch does not show expansion, which fits with the described distribution of Notch and its ligands. It would be also worthwhile to highlight the special situation for the mandibular arch (which is only patterned in its most caudal part by the patterning system described here) in the discussion
(section 440 ff)
- 449: I assume the authors mean “homogeneous” (not “homologous”)
- 515: add “to” in front of “the situation”
- Fig. 1: c1 and c2 not explained in legend
- Fig. 3: there are no black and white arrowheads as mentioned in legend
- Fig. 4: instead of the arrowheads and arrows described in the legend there are open and filled arrowheads. I am not convinced by the results shown in Fig. 4 C; there does not seem to be a clear expansion of the rostral marker Vgll2, in particular for the mandibular arch
- Fig. 5: there is no asterisk; labels P and G need to be explained in legend
- Fig. 6: title of legend should read “... illustrating how Notch ...”
- in Table S3, some of the references given for in situ probes are for Xenopus papers (Pandur and Moody, 2000; David et al., 2001). I assume mouse probes have been used and the proper papers should be cited; many of the references listed here are not cited in the reference list

Reviewer 2

Advance summary and potential significance to field

In this manuscript the authors investigate subdivision of the epibranchial territory into individual placodes (geniculate, petrosal and nodose). The authors define the timing of epibranchial placode

© 2020. Published by The Company of Biologists under the terms of the Creative Commons Attribution License (https://creativecommons.org/licenses/by/4.0/).

formation in mouse, identify molecular markers that accompany this process and for the first time describe distinct anterior and posterior regions within each epibranchial placode territory with respect to the branchial clefts. Finally, they identify Notch signalling that mediates the segregation of anterior-posterior regions.

Comments for the author

Overall, this is a nice study that reveals so far unidentified patterning of the epibranchial territory and a molecular mechanism that controls the anterior-posterior subdivision of each placode region. The data are well presented supporting the authors' conclusions and are of interest to the placode field.

To provide more mechanistic insight it would be interesting to define the Notch targets and how they interact with the molecular markers that define anterior-posterior regions, whether they are direct targets or not and to evaluate whether factors like Vgll2 and Irx5 play a role in setting up these domains.

I would suggest that the authors avoid the term 'compartments'; this is a very clearly defined term that implies lineage restriction within a territory, and there is no evidence for true compartments in the epibranchial territory.

The title implies that Notch signalling controls differentiation, however the manuscript does not address differentiation in the epibranchial placodes.

Reviewer 3

Advance summary and potential significance to field

In this manuscript, Wang and colleagues investigate the process involved in the formation of the 3 epibranchial placodes from the common posterior placodal area (PPA). They have identified early patterning events in the pharyngeal arch (PA) ectoderm, and characterized the molecular signature of the anterior half (Sox2+/Fgf3+/Etv5+) posterior half (Vgll2+s/Irx5+) of the PA ectoderm that participates in the establishment of the placodal neurogenic domains. Changing the boundary of expression of these genes by modulating Notch signaling impact the formation of these placodes. The study makes an interesting contribution to the field.

Comments for the author

The work is largely descriptive and provides only minimal insights into the molecular mechanisms underlying the processes governing epibranchial placode development. The experiments testing a potential role of Notch signaling in regulating these processes are not sufficiently developed to conclude that Notch signaling is involved in epibranchial patterning, segregation and differentiation.

The major concern is that the functional analysis (activation and inhibition of Notch Signaling; Fig 4) is limited to a single embryonic stage (E9.5), which fails to capture the dynamic nature of epibranchial placode development. Without a more precise analysis it is difficult to assign a specific function to Notch signaling in the formation of these placodes. The authors need to expand their study to include a broader range of embryonic stages covering the different phases of epibranchial placode development. Also, as discussed in the introduction, the establishment of adjacent regions of apoptosis and proliferation is critical to orchestrate the development of these placodes, and as such it would be important to determine whether these processes are affected in these animals.

Other issues:

Figure 2A – The position of the clefts should be indicated, and higher magnification views of the areas of interest should be provided, especially at early stages. The expression of Sox2 and Neurogenin2 is not described in the text. For panel 2B the text indicates that the 3 clefts appear sequentially, however only 2 are indicated.

© 2020. Published by The Company of Biologists under the terms of the Creative Commons Attribution License (https://creativecommons.org/licenses/by/4.0/).

Figure 3 – The text states that Notch1 is expressed in the caudal ectoderm of each cleft, there is no staining associated with c2 on the image provided (Fig 3J).

Figure 4 – The reported reduction in neurog2 expression upon Notch signaling inhibition is a little surprising considering the known function of Notch signaling as a negative regulator of neuronal differentiation. In fact it looks like neurog2 expression is shifted anteriorly rather than reduced (Fig 4L). The authors do not adequately discuss this result. The authors state that “Vgll2 was specifically lost in Pax2-Cre;Rosa embryos while endodermal expression was retained”, however Vgll2 is not detected in the PA endoderm in WT embryos.

On page 5 the authors state: “We identify an early patterning event, with the appearance of a rostral Vgll2+/Irx5+ domain and a caudal Sox2+/Fgf3+/Etv5+ domain located on opposite sides of the first pharyngeal cleft.” On page 15 they state: “As such each proximal PA, the anterior half was covered with Sox2+/Fgf3+/Etv5+ ectodermal cells, whereas the posterior half was Vgll2+/Irx5+.” This can be confusing and I would suggest that the authors use a unified terminology to describe these two domains throughout the manuscript, either in reference to the PA or to the pharyngeal cleft.

For developmental stages, the authors are constantly switching between embryonic day (E) and somite stage (ss). This is unnecessarily confusing and I would recommend that the authors use one or the other.

The discussion is very lengthy and should be more concise.

The figure legends are cursory, and often do not match the panels. They will require substantial editing. What is the purpose of the asterisk in Fig 1I? The legend of Fig 3 mentions white and black arrowheads that are not present on the images. For Fig 3 and Fig 5 the size of the scale bars are not indicated. The legend of Fig 5 states that the caudal (c) and rostral (r) domains relative to each cleft are indicated, but this is not the case.
These are just a few examples.

First revision

Author response to reviewers' comments

20th December, 2019.

Dear Francois,

We thank you and the reviewers for your constructive comments on our work and for the possibility to resubmit a revised version. We are also grateful for the specific comments from your side, which have been a useful guide in preparing the revised version.

“Editor’s comment:

As you will see, all the referees express great interest in your work, but they also have some significant criticisms and recommend a substantial revision of your manuscript before we can consider publication. If you are able to revise the manuscript along the lines suggested, which may involve further experiments, I will be happy to receive a revised version of the manuscript. The identification of the Notch targets mentioned by referee 2 would be of course interesting but seems beyond the scope of the study unless you already have some evidence in that direction. The request of referee 3 that you analyse the effect of manipulations of Notch signalling at other stages than E9.5 will also require a significant amount of work without being necessarily informative. Instead, during the post-review discussion, referees asked that you define the time point when each Cre activates or inhibits Notch in the epibranchial territory and interpret the phenotype accordingly,

© 2020. Published by The Company of Biologists under the terms of the Creative Commons Attribution License (https://creativecommons.org/licenses/by/4.0/).

i.e. if there is only a short time between Notch modulation and phenotype analysis, one could argue for an immediate effect. I agree this would be a valuable addition to your study."

We appreciate these comments and the guidance for how to revise the manuscript. We have addressed these points and now report on the timeline for Cre activation in the LOF and GOF Notch experiments. Using the *Pax2-Cre* drivers crossed with *Rosa*$^{N1\text{-}IC}$ fluorescent reporter lines (N1ICD is linked to GFP via an IRES), we showed that GFP signal (indicating N1ICD expression) could be detected from E8.5 till E9.5 in the epibranchial placodal regions (see Figure A appended). We had analysed the expression of *Vgll2* and *Fgf3* in *Pax2-Cre;Rosa*$^{N1\text{-}IC}$ mutants at E9.0, and we found that *Fgf3* expression was increased and *Vgll2* expression was reduced in *Pax2- Cre;Rosa*$^{N1\text{-}IC}$ mutants (Figure A), suggesting an immediate effect of Notch activation in the epibranchial territory.

As *Actin-Cre* is active from zygotic stage, *Actin-Cre;Rbpj*$^{f/f}$ could be interpreted as a null mutant and our analysis indeed showed that Rbpj protein was completely absent in an E9.5 embryo (Figure B). We analysed the phenotype of *Actin-Cre;Rbpj*$^{f/f}$ mutant at E8.5, and showed that Sox2 and Etv5 expression were not affected in *Actin-Cre; Rbpj*$^{f/f}$ embryos at E8.5 (Figure B), whereas their expression was reduced in *Actin-Cre; Rbpj*$^{f/f}$ embryos at E9.5 (Fig. 4). Thus, the effect of the Notch signalling inhibition was not observed until E9.5. We had also examined *Pax2-Cre;Rbpj*$^{f/f}$ mutant, however, the expression of rostral or caudal domain genes as well as the differentiation of neurons were not affected (Figure B), possibly due to the late onset of *Pax2-Cre* expression. Therefore, only when *Rbpj* was knocked-out from zygotic stage, the LOF effect on the epibranchial placode was noted.

The comments from the reviewers are detailed in the point-by-point response below. We feel that the additional experiments and rewritings have led to a significantly stronger manuscript, which we believe will meet the standards for publication in Development.

"Reviewer 1 Advance Summary and Potential Significance to Field:

This study addresses how the epibranchial placodes, which contribute viscerosensory neurons to the cranial PNS, are patterned and segregate from the other during early mouse embryonic development. The authors first establish that the Pax2 expressing posterior placodal area (PPA) gives rise to the otic placode, epibranchial placodes as well as the epidermal regions in between and that the discrete epibranchial placodes form by the thinning out of these intervening epidermal domains. They then show that the regions of the PPA rostral and caudal to the pharyngeal clefts express different marker genes including several ligands and receptors of the Notch pathway. Finally, the authors use Notch gain and loss of function mouse mutants to show that Notch signalling plays an important role in the patterning of the PPA (with high Notch promoting rostral-to -cleft identity and low Notch promoting caudal-to-cleft identity). The experiments described in this paper are straightforward and the results obtained overall well documented (see detailed comments below). The study makes an important contribution to our understanding of early ectodermal patterning in the vertebrate embryo. It is, therefore, of wide interest and suitable for publication in Development after some relatively minor concerns are addressed."

We thank the reviewer for these positive comments on the importance of our study.

"Reviewer 1 Comments for the Author:

Detailed comments:
-128: here the authors need to make clearer that their use of "rostral" and "caudal" throughout the paper is meant in relation to the pharyngeal clefts (it is mentioned in passing but should be stressed more). I was initially confused and would have found it more appropriate to talk about the rostral and caudal part of each pharyngeal arch (in which case the "caudal" domains of the authors become "rostral") since this is a more obvious anatomical unit. In the figure legends it would also be useful to explicitly write "rostral to the cleft" etc to avoid confusion. Also, the pharyngeal clefts are not always well visible in all figures. I suggest to mark the pharyngeal clefts clearly in all figs (that would be clearer than marking rostral and caudal expression domains with different arrowheads)"

© 2020. Published by The Company of Biologists under the terms of the Creative Commons Attribution License (https://creativecommons.org/licenses/by/4.0/).

We appreciate this comment and realize that we were not very clear with regard to the nomenclature. In the revised version, we have clarified our use of "rostral" and "caudal" and their relation to the pharyngeal cleft (Results, line 217). Furthermore, we have added a schematic diagram in Fig. 1A to explain these terms already at the beginning of the manuscript. We have also changed the figure legends in line with the comment, the pharyngeal clefts are clearly marked, and rostral and caudal expression domains are marked with different arrowheads in all figures in the revised version.

"-285: "Vgll2 expanded into the caudal domains". This should be described in a bit more detail (1) in emphasizing that this is a rostral expansion into the ectoderm located caudal to a more anterior cleft) and (2) in explicitly describing, which pharyngeal arches are affected. It is notable that this is not observed for the mandibular arch: i.e. the rostral domain of the first pharyngeal pouch does not show expansion, which fits with the described distribution of Notch and its ligands. It would be also worthwhile to highlight the special situation for the mandibular arch (which is only patterned in its most caudal part by the patterning system described here) in the discussion (section 440 ff)"

This is a good point. We provide a more precise description of the *Vgll2* expansion and describe which pharyngeal clefts are affected (Results, line 299). We also specifically indicate that *Vgll2* expansion is not observed in the mandibular arch (Results, line 300-302), and we thank the reviewer for pointing this out in relation to the expression of Notch receptors and ligands. We have revised our discussion that the rostrocaudal patterning only includes a limited region derived from the PPA, rostral to the first pharyngeal cleft, while the rest of the mandibular arch was not affected (Discussion, line 454-456).

"-449: I assume the authors mean "homogeneous" (not "homologous")
-515: add "to" in front of "the situation"
-Fig. 1: c1 and c2 not explained in legend
-Fig. 3: there are no black and white arrowheads as mentioned in legend
-Fig. 4: instead of the arrowheads and arrows described in the legend, there are open and filled arrowheads. I am not convinced by the results shown in Fig. 4 C; there does not seem to be a clear expansion of the rostral marker Vgll2, in particular for the mandibular arch
-Fig. 5: there is no asterisk; labels P and G need to be explained in legend
-Fig. 6: title of legend should read "... illustrating how Notch ..."
-in Table S3, some of the references given for in situ probes are for Xenopus papers (Pandur and Moody, 2000; David et al., 2001). I assume mouse probes have been used and the proper papers should be cited; many of the references listed here are not cited in the reference list"

All the above points have been addressed in the revised version:.
-494: "homologous" was changed to "homogeneous" (line 451)
-511: sentence structure was adjusted (line 511)
-Fig.1: all the annotations (including c1 and c2) were explained in the figure legend (line 634-636)
-Fig. 3: In our revised Fig. 3, we used filled and open arrowheads to indicate rostral and caudal gene expression, they were also aligned with figure legends (line 658-660)
-Fig. 4: the indicating arrowheads were cleaned up in the revised version. Besides, we also explained in the revised manuscript that rostral patterning of the first pharyngeal cleft did not include mandibular arch (lines 203-204, 300-302, 454-456), which is consistent with the expression pattern with Notch factors (line 301-302).
-Fig. 5: asterisks were added to indicate ectopic Neurog2 expression, P and G were explained in figure legend as well (line 687-688)
-Fig. 6: title of legend was revised (line 690-692)
-Table S3: all the probe references were checked, updated and cited in the supplementary information

"Reviewer 2 Advance Summary and Potential Significance to Field:

In this manuscript the authors investigate subdivision of the epibranchial territory into individual placodes (geniculate, petrosal and nodose). The authors define the timing of epibranchial placode formation in mouse, identify molecular markers that accompany this process and for the first time describe distinct anterior and posterior regions within each epibranchial placode territory with

© 2020. Published by The Company of Biologists under the terms of the Creative Commons Attribution License (https://creativecommons.org/licenses/by/4.0/).

respect to the branchial clefts. Finally, they identify Notch signalling that mediates the segregation of anterior- posterior regions.

Reviewer 2 Comments for the Author:

Overall, this is a nice study that reveals so far unidentified patterning of the epibranchial territory and a molecular mechanism that controls the anterior-posterior subdivision of each placode region. The data are well presented supporting the authors' conclusions and are of interest to the placode field."

We thank the reviewer for these positive comments on our study.

"To provide more mechanistic insight it would be interesting to define the Notch targets and how they interact with the molecular markers that define anterior-posterior regions, whether they are direct targets or not and to evaluate whether factors like Vgll2 and Irx5 play a role in setting up these domains."

This is a good suggestion. To try to provide more insights into Notch signalling pathway, we assessed the expression of Notch ligands (*Jag1*, *Jag2* and *Dll1*) and possible downstream targets (*Hey1*, *HeyL* and *Hes6*) in *Pax2-Cre;Rosa*^{NICD} and *Actin- Cre;Rbpj*$^{f/f}$ mutants (Fig. 4A). These data suggested a correlation between *Jag1* with the caudal domain, *Jag2* with the rostral domain and *Dll1* with the neurogenic domain, and these ligands possibly regulate downstream activities via *Hey1* and *HeyL*. In Fig. 6B, we also included a schematic diagram to illustrate the expression of Notch signalling factors in the placodal cells in the rostral, caudal and dorsal-neural regions.

We have also elaborated on the possible direct or indirect relationship between the level of Notch signalling and the expression of genes that are up- or downregulated in the GOF and LOF situations. We have analysed by ChIP-seq in a cell line (MDA-MB-231) by ChIP-seq for Rbpj (CSL) whether CSL binds in a Notch-responsive manner to CSL binding sites within or in the vicinity of the genes upregulated by Notch (*Fgf3* and *Sox2*) and the genes that are downregulated (*Vgll2* and *Irx5*) (Figure C and D, respectively). Our data reveal that there is no CSL-binding to putative regulatory regions for these genes that is regulated by altering the level of Notch signalling, while this is the case for a control Notch target gene (*Hes4*) (Figure C,D below). While conducted in a setting which admittedly is not identical to the epibranchial placodes, we believe this argues that the Notch effects on *Fgf3*, *Sox2*, *Vgll2* and *Irx5* expression are indirect, and we briefly mention this in the revised version (Discussion, line 521- 527). As regards *Hes1* as a possible mediator of the negative regulation of *Vgll2* and *Irx5* expression, we have also bioinformatically looked for Hes1 binding sites in and around the *Vgll2* and *Irx5* genes, but since Hes1 binding sites are rather loosely defined, there are multiple potential binding sites. The few ChIP-seq studies that have been conducted for Hes1 DNA-binding also do not provide a clear picture as to whether one or more of these sites are functional. In the light of this, we just mention the possibility that Hes1 (or other Hes/Hey genes) may be involved in negatively regulating *Vgll2* and *Irx5* expression (Discussion, line 518-523) (see also our response to Reviewer 1).

To address the specific roles of *Vgll2* and *Irx5* through genetic experiments in the mouse is indeed a good suggestion, but we do not have the required floxed mouse models at our disposal. In line with the Editor's comments above, we therefore think that these experiments would be better conducted in a separate follow-up study.

Finally, and as discussed above, we have improved the analysis of the Cre models used to regulate Notch signalling. The *Pax2-Cre* driver produces Cre activity from E8.5 till E9.5 in the epibranchial placodal regions (Figure A), while the *Actin-Cre* is active from the zygotic stage (Figure B).

"I would suggest that the authors avoid the term 'compartments'; this is a very clearly defined term that implies lineage restriction within a territory, and there is no evidence for true compartments in the epibranchial territory."

We appreciate this comment, and we now refrain for defining the areas as compartments. Instead, we call these as regions or domains in the revised manuscript.

© 2020. Published by The Company of Biologists under the terms of the Creative Commons Attribution License (https://creativecommons.org/licenses/by/4.0/).

“The title implies that Notch signalling controls differentiation, however the manuscript does not address differentiation in the epibranchial placodes.”

The reviewer is correct, and we agree that the title was not optimal and tried to cover too much ground. We have thus removed the term differentiation from the title in the revised version.

“Reviewer 3 Advance Summary and Potential Significance to Field:

In this manuscript, Wang and colleagues investigate the process involved in the formation of the 3 epibranchial placodes from the common posterior placodal area (PPA). They have identified early patterning events in the pharyngeal arch (PA) ectoderm, and characterized the molecular signature of the anterior half (Sox2+/Fgf3+/Etv5+) posterior half (Vgll2+s/Irx5+) of the PA ectoderm that participates in the establishment of the placodal neurogenic domains. Changing the boundary of expression of these genes by modulating Notch signaling impact the formation of these placodes. The study makes an interesting contribution to the field.”

We thank the reviewer for the positive comments on our work.

“Reviewer 3 Comments for the Author:

The work is largely descriptive and provides only minimal insights into the molecular mechanisms underlying the processes governing epibranchial placode development. The experiments testing a potential role of Notch signaling in regulating these processes are not sufficiently developed to conclude that Notch signaling is involved in epibranchial patterning, segregation and differentiation.“

We agree that we have not yet a complete understanding of the role of Notch signalling in the decision-making for epibranchial placodes (see also our response to Reviewer 2), but we respectfully disagree that we do not provide evidence for a role of Notch in the process. We believe that the LOF and GOF data for Notch signalling (by ablating *Rbpj* and expressing *N1ICD*, respectively) provide evidence that Notch is implicated, and importantly that LOF and GOF of Notch provide opposing phenotypes (Fig. 4A). We have however tried to improve the analysis in several ways. Thus, in the revised version, we provide a better time axis for Cre activation and assess whether *Vgll2* and *Irx5* are direct or indirect Notch targets (see also response to Reviewer 2’s comments). As discussed above, we have also analysed by ChIP-seq whether the upregulated (*Fgf3*, *Sox2*) or downregulated (*Vgll2*, *Irx5*) genes are direct or indirect target genes, i.e. whether there is Notch-regulated CSL-binding to CSL binding sites in or near the genes. In cell line experiments, we do not find evidence for this, while a Notch downstream gene (*Hes4*) is indeed regulated (Figure C,D below). We agree that the ChIP-seq experiment is done in a cell line-based system, but we anyway briefly mention in the revised version that effects may be indirect. As also discussed above, we have performed a more deep-drilling analysis of how other genes in the Notch pathway (ligands and *Hes*/*Hey* genes) are regulated in the GOF and LOF situation (see also our response to Reviewer 2).

“The major concern is that the functional analysis (activation and inhibition of Notch Signaling; Fig. 4) is limited to a single embryonic stage (E9.5), which fails to capture the dynamic nature of epibranchial placode development. Without a more precise analysis it is difficult to assign a specific function to Notch signaling in the formation of these placodes. The authors need to expand their study to include a broader range of embryonic stages covering the different phases of epibranchial placode development.”

This point of ‘single time point’ or ‘inclusion of a broader temporal regulation’ has also been raised by Reviewer 2 and summarized by the Editor, please refer to our response to the Editor and to Reviewer 2 above. We tried to provide a precise *Pax2-Cre* activation timeline to show the activation of NICD in the mutant (Figure A). Besides, since *Rbpj* is deleted at the zygote stage, we also showed the disrupted development as well as the deletion of Rbpj by antibody staining in Figure B. Moreover, we have also addressed the relationship between Notch and the up- and downregulated genes in more detail (Figure C and D, see also discussion above).

© 2020. Published by The Company of Biologists under the terms of the Creative Commons Attribution License (https://creativecommons.org/licenses/by/4.0/).

"Also, as discussed in the introduction, the establishment of adjacent regions of apoptosis and proliferation is critical to orchestrate the development of these placodes, and as such it would be important to determine whether these processes are affected in these animals."

This is a very good suggestion. We have now analysed apoptosis by TUNEL staining and proliferation by PH3 and CyclinD1 staining (data are shown in Fig. 1 M-O and in Supplementary Figure S3). In wildtype controls, apoptosis is mainly observed in cells at the first pharyngeal cleft, which is consistent with previous studies (Washausen and Knabe, 2013; Washausen et al., 2005), and proliferation revealed by PH3 staining distributed evenly along the pharyngeal ectoderm. Comparing *Pax2-Cre;Rosa*^{NICD} sample with WT reveals that apoptosis and proliferation are not changed. In the *Actin- Cre;Rbpj*$^{f/f}$ embryos, more apoptosis and less proliferation could be observed in both rostral and caudal domains. These data suggested that the expanded rostral characteristic in *Actin-Cre;Rbpj*$^{f/f}$ was unlikely due to the proliferation of rostral domain or the apoptosis of the caudal domain. For instance, the expression of CyclinD1 is normally restricted to the caudal domain (Fig. 1M). We also analysed CyclinD1 expression in the Notch GOF and LOF mutants (Fig. 4B). The data here reinforce the notion that CyclinD1 expression is associated with the caudal territory.

"Other issues:
Figure 2A – The position of the clefts should be indicated, and higher magnification views of the areas of interest should be provided, especially at early stages. The expression of Sox2 and Neurogenin2 is not described in the text. For panel 2B the text indicates that the 3 clefts appear sequentially, however only 2 are indicated."

We appreciate this comment and agree that the position of the branchial clefts was not very well described. For panel 2B, limited by the section panel, very few sections could capture the cleft1 and cleft3 simultaneously. We now have replaced the images to show all three clefts with three rostral and three caudal domains. We have marked all three pharyngeal clefts in panel 2B and pointed out the expression of rostral and caudal genes with arrowheads in panel 2A. We also described the expression pattern of *Sox2* and *Neurog2* in the revised manuscript (line 213-216 and 226-228).

"Figure 3 – The text states that Notch1 is expressed in the caudal ectoderm of each cleft, there is no staining associated with c2 on the image provided (Fig 3J)."

We have revised the figures and provided data to show the expression of Notch 1 in the ectodermal cells caudal to the 1st and 2nd clefts in Fig. 3B. Moreover, we have previously reported the expression of Notch 1 in the epibranchial epithelial cells caudal to the clefts in Zhang et al. 2017.

"Figure 4 – The reported reduction in neurog2 expression upon Notch signaling inhibition is a little surprising, considering the known function of Notch signaling as a negative regulator of neuronal differentiation. In fact, it looks like neurog2 expression is shifted anteriorly rather than reduced (Fig 4L). The authors do not adequately discuss this result. The authors state that "Vgll2 was specifically lost in Pax2-Cre;Rosa embryos, while endodermal expression was retained", however Vgll2 is not detected in the PA endoderm in WT embryos."

We thank the reviewer for pointing this out. According to the expression pattern of *Neurog2* in the *Actin-Cre;Rbpj*$^{f/f}$ mutant, *Neurog2* expression was observed dorsal to the first arch, likely induced from the trigeminal ganglia and not from the epibranchial placodes. The expression of *Neurog2* in trigeminal ganglia was indeed increased in the *Actin-Cre;Rbpj*$^{f/f}$ embryos (Fig. 4A). However, in the epibranchial placodes, the expression of *Neurog2* is reduced, as compared to wildtype controls (Fig. 4A). It is likely that Notch signalling here not only regulates neural differentiation, but also controls the initial production of caudal progenitors.

Regarding the expression of *Vgll2* in the pharyngeal endoderm, we found that *Vgll2* was consistently expressed in the pharyngeal endoderm around the developing pouches from E8.5 to E9.5 (see Fig. 2B), though the expression level in the endoderm was much lower in E9.5 wildtype embryos (Fig. 2B). The main point was that in the *Pax2-Cre;Rosa*^{NICD} embryos, the ectodermal expression of *Vgll2* was indeed specifically lost (Fig. 4B). We have revised this statement in the revised manuscript (Results, line 296-298).

© 2020. Published by The Company of Biologists under the terms of the Creative Commons Attribution License (https://creativecommons.org/licenses/by/4.0/).

"On page 5 the authors state: "We identify an early patterning event, with the appearance of a rostral Vgll2+/Irx5+ domain and a caudal Sox2+/Fgf3+/Etv5+ domain located on opposite sides of the first pharyngeal cleft." On page 15 they state: "As such each proximal PA, the anterior half was covered with Sox2+/Fgf3+/Etv5+ ectodermal cells, whereas the posterior half was Vgll2+/Irx5+." This can be confusing, and I would suggest that the authors use a unified terminology to describe these two domains throughout the manuscript, either in reference to the PA or to the pharyngeal cleft."

We agree that this section was not well written and difficult to follow. We have added a schematic diagram in the revised Figure 1A to illustrate the relative position of rostral and caudal domains to each pharyngeal cleft, and used the terminology of anterior and posterior exclusively for pharyngeal arches.

"For developmental stages, the authors are constantly switching between embryonic day (E) and somite stage (ss). This is unnecessarily confusing and I would recommend that the authors use one or the other."

We apologize for the confusion and inconsistency in the original version. We now throughout the manuscript and figures use embryonic day (E) as the primary nomenclature and complement it with the somite stage in brackets when dealing with dynamic changes of gene expression (e.g. Fig. 2A). In this way, we hope that the paper will be easier to read.

"The discussion is very lengthy and should be more concise."

We agree and we have condensed the discussion considerably in the revised manuscript.

"The figure legends are cursory, and often do not match the panels. They will require substantial editing. What is the purpose of the asterisk in Fig 1I? The legend of Fig 3 mentions white and black arrowheads that are not present on the images. For Fig 3 and Fig 5 the size of the scale bars are not indicated. The legend of Fig 5 states that the caudal (c) and rostral (r) domains relative to each cleft are indicated, but this is not the case. These are just a few examples."

We appreciate that the reviewer alerted us to these shortcomings in the Figure legends. We have corrected the mistakes pointed out and also generally reworked the Figure legends to make them better aligned with the actual figures.

In conclusion, we thank the reviewers for fair and constructive comments, and for suggestions for how to improve the manuscript.

Yours sincerely,

Mai Har Sham

Figures appended:
Figure A. Temporal analysis of activation of N1-ICD by Pax2-Cre
Figure B. Knock-out of *Rbpj* by Actin-Cre and Pax-Cre in the posterior placodal area Figure C. CSL (Rbpj) ChIP-Seq analysis of *Vgll2* and *Irx5* on MDAMB231 cells Figure D. CSL (Rbpj) ChIP-Seq analysis of *Fgf3* and *Sox2* on MDAMB231 cells

© 2020. Published by The Company of Biologists under the terms of the Creative Commons Attribution License (https://creativecommons.org/licenses/by/4.0/).

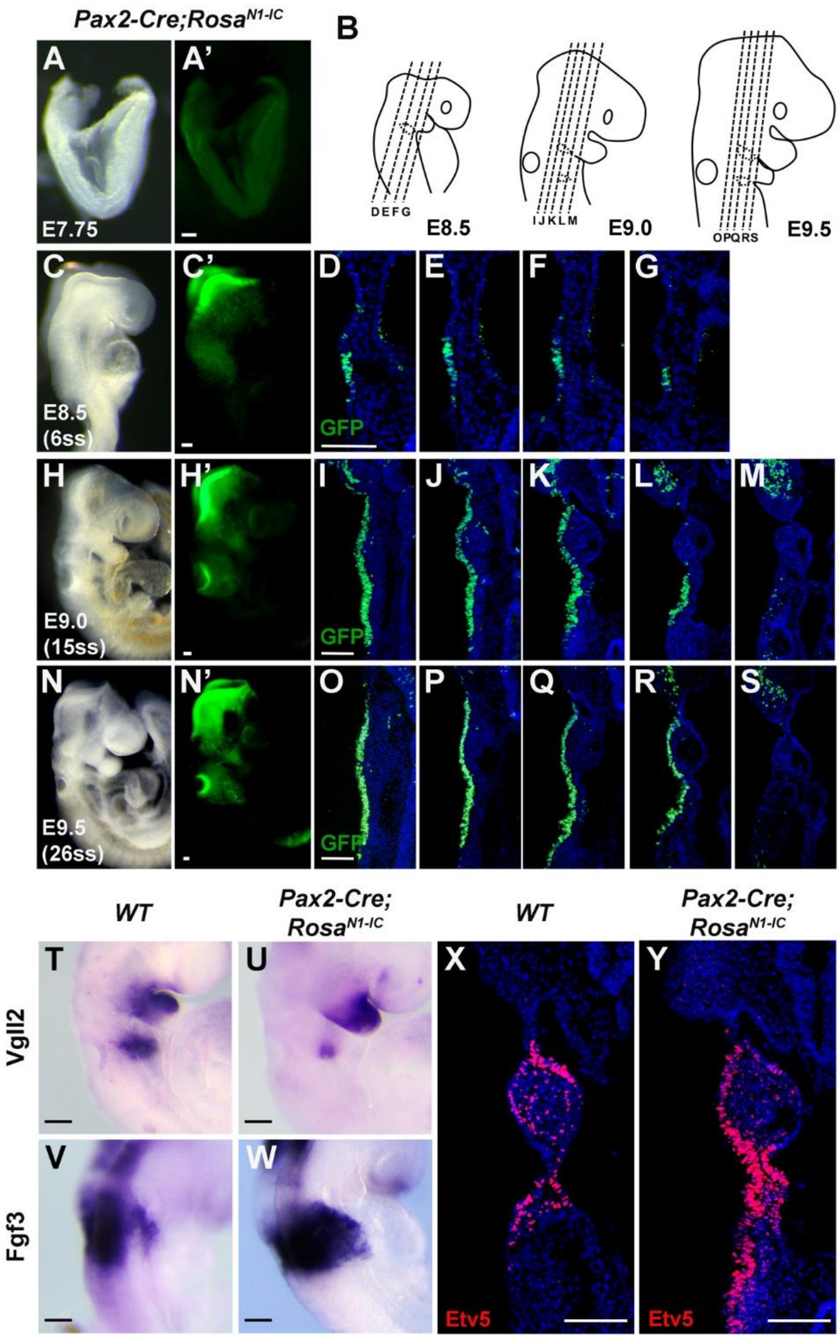

Figure A. Temporal analysis of Notch activation in the proximal pharyngeal ectoderm using Pax2-Cre. (A-S) Whole mount bright field and fluorescent images, and immunostaining for GFP on serial coronal sections of *Pax2-Cre;Rosa^{N1-IC}* embryos at indicated stages. (n=3) Planes of sections are indicated as dashed lines in (B). Note that N1ICD is activated in the proximal pharyngeal epithelium from E8.5. (T-W) Whole mount *in situ* hybridization showing reduced *Vgll2* (U) and expanded *Fgf3* (W) expression on *Pax2-Cre;Rosa^{N1-IC}* embryos compared with *WT* at E9.0 (n=3). (X-Y) Immunostaining for Etv5 on coronal sections of E9.0 embryos showing expanded ectoderm expression to rostral domain in *Pax2-Cre;Rosa^{N1-IC}* (Y) compared with *WT*. (n=3) Scale bar = 100 µm.

© 2020. Published by The Company of Biologists under the terms of the Creative Commons Attribution License (https://creativecommons.org/licenses/by/4.0/).

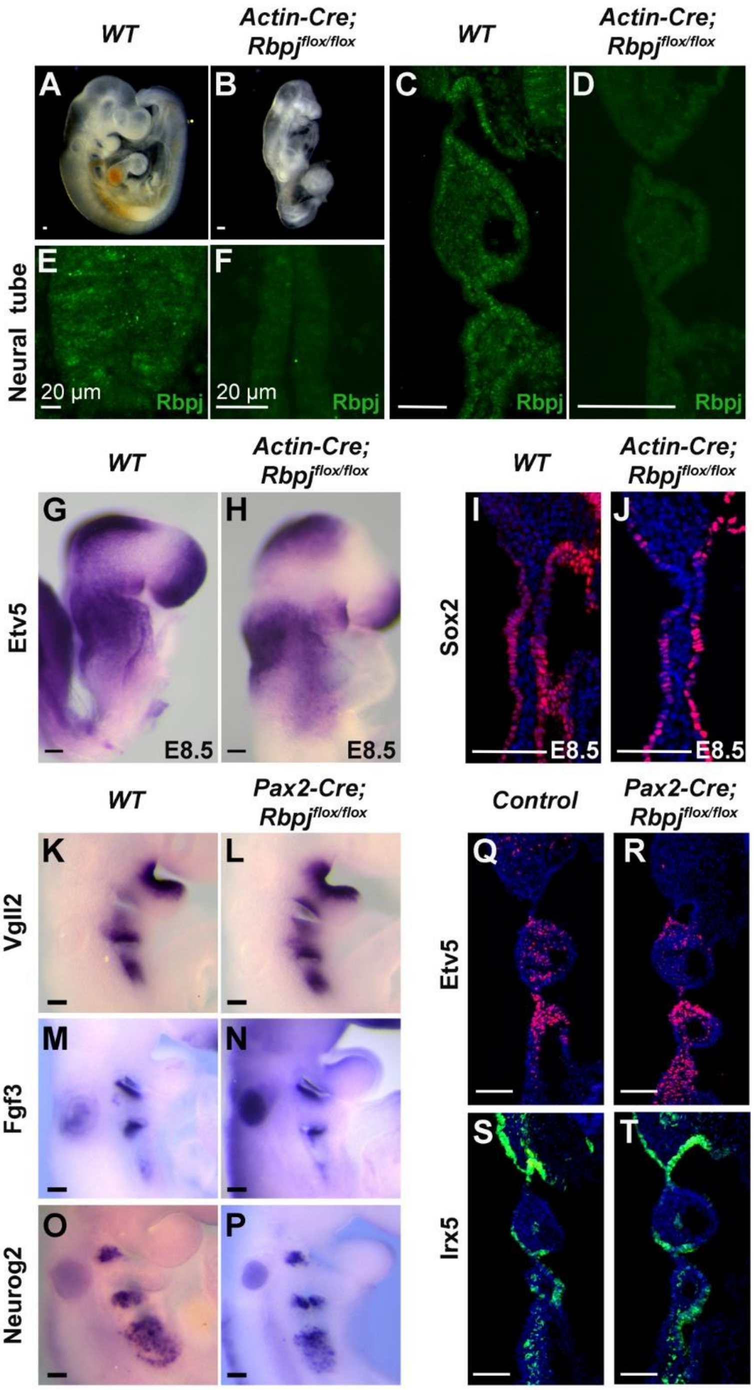

Figure B. Knock-out of *Rbpj* by *Actin-Cre* and *Pax2-Cre* in the posterior placodal area. (A-B) Whole mount image of *WT* and *Actin-Cre;Rbpj*$^{flox/flox}$ embryos at E9.5 (n≥3). (C-F) Immunostaining of *Rbpj* in pharyngeal arch region (C and D) and in neural tube (E and F) in *WT* and *Actin-Cre;Rbpj*$^{flox/flox}$ embryos at E9.5 showing loss of Rbpj expression. (n=1) (G-J) Whole mount *in situ* hybridization and immunostaining on coronal sections of *WT* and *Actin-Cre;Rbpj*$^{flox/flox}$ embryos at E8.5 showing no change of Sox2 (J) and Etv5 (H) expression in the mutants at this stage. (n≥2). (K-T) Whole mount *in situ* hybridization (K-P) and immunostaining on coronal sections (Q-T) of *WT* and *Pax2-Cre;Rbpj*$^{flox/flox}$ embryos at E9.5 (n≥3) for indicated genes, showing no change in gene expression patterns in the mutant embryos. Scale bar = 100 µm.

© 2020. Published by The Company of Biologists under the terms of the Creative Commons Attribution License (https://creativecommons.org/licenses/by/4.0/).

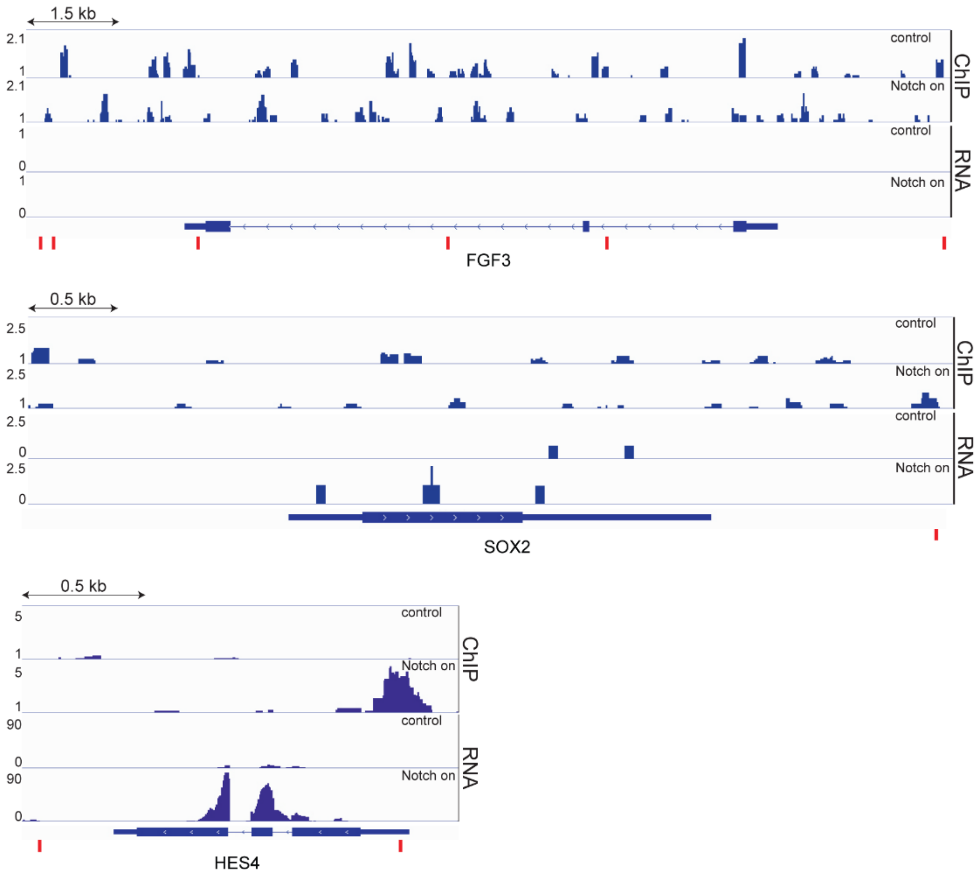

Figure C. CSL (RBPJ) ChIP-seq performed on chromatin isolated from MDAMB231 cells. Visualisation of CHIP-seq and RNA reads around the genomic loci of FGF3, SOX2 and HES4. Cells were either cultivated with Jagged1-coated plates for 48h (Notch on) or left untreated (control). The red bars in the genomic loci tracks indicate the position of a consensus CSL (RBPJ) binding motif (GTGGGAA), which in the case of HES4 coincides with the location of the promoter.

© 2020. Published by The Company of Biologists under the terms of the Creative Commons Attribution License (https://creativecommons.org/licenses/by/4.0/).

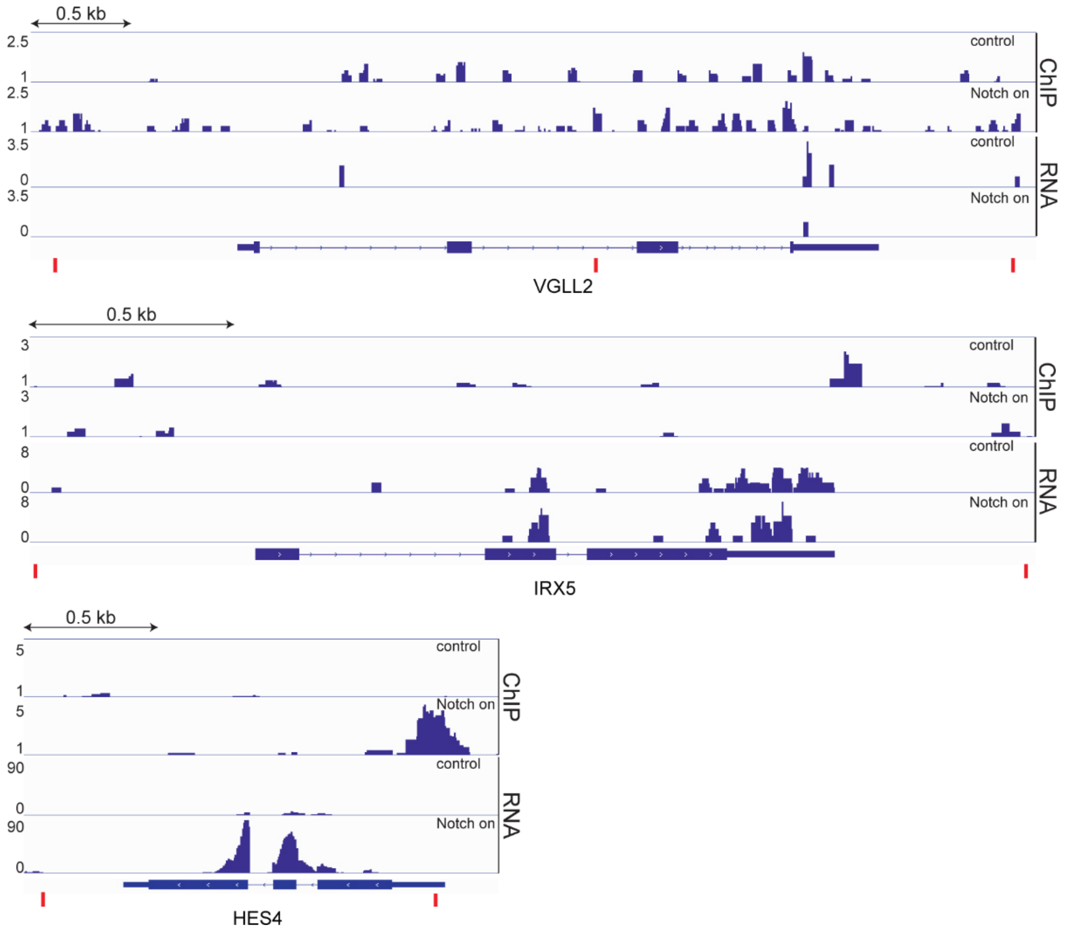

Figure D. CSL (RBPJ) ChIP-seq performed on chromatin isolated from MDAMB231 cells. Visualisation of CHIP-seq and RNA reads around the genomic loci of VGLL2, IRX5 and HES4. Cells were either cultivated on Jagged1-coated plates for 48h (Notch on) or left untreated (control). The red bars in the genomic loci tracks indicated the position of a consencus CSL (RBPJ) binding motif (GTGGGAA), which in the case of HES4 coincides with the location of promoter.

<u>Second decision letter</u>

MS ID#: DEVELOP/2019/183665

MS TITLE: Notch signalling regulates epibranchial placode patterning and segregation

AUTHORS: Li Wang, Junjie Xie, Haoran Zhang, Long Hin Tsang, Sze Lan Tsang, Eike-Benjamin Braune, Urban Lendahl, and Mai Har Sham
ARTICLE TYPE: Research Article

I am delighted to tell you that your manuscript has been accepted for publication in Development, pending our standard ethics checks.

<u>Reviewer 2</u>

Advance summary and potential significance to field

In this manuscript the authors investigate subdivision of the epibranchial territory into individual placodes (geniculate, petrosal and nodose). The authors define the timing of epibranchial placode

© 2020. Published by The Company of Biologists under the terms of the Creative Commons Attribution License (https://creativecommons.org/licenses/by/4.0/).

formation in mouse, identify molecular markers that accompany this process and for the first time describe distinct anterior and posterior regions within each epibranchial placode territory with respect to the branchial clefts. Finally, they identify Notch signalling that mediates the segregation of anterior-posterior regions.

Comments for the author

The revised version of the manuscript has considerably improved and the authors have addressed the previous comments.

© 2020. Published by The Company of Biologists under the terms of the Creative Commons Attribution License (https://creativecommons.org/licenses/by/4.0/).
